# Microfluidic Giant Polymer Vesicles Equipped with Biopores for High‐Throughput Screening of Bacteria

**DOI:** 10.1002/advs.202307103

**Published:** 2023-12-29

**Authors:** Lukas Heuberger, Daniel Messmer, Elena C. dos Santos, Dominik Scherrer, Emanuel Lörtscher, Cora‐Ann Schoenenberger, Cornelia G. Palivan

**Affiliations:** ^1^ Department of Chemistry University of Basel Mattenstrasse 22 Basel 4002 Switzerland; ^2^ IBM Research Europe–Zürich Säumerstrasse 4 Rüschlikon 8803 Switzerland; ^3^ NCCR‐Molecular Systems Engineering Mattenstrasse 24a, BPR 1095 Basel 4058 Switzerland; ^4^ Swiss Nanoscience Institute (SNI) University of Basel Klingelbergstrasse 82 Basel 4056 Switzerland

**Keywords:** antibiotics, bacteria, GUVs, high‐throughput screening, polymers, vesicles

## Abstract

Understanding the mechanisms of antibiotic resistance is critical for the development of new therapeutics. Traditional methods for testing bacteria are often limited in their efficiency and reusability. Single bacterial cells can be studied at high throughput using double emulsions, although the lack of control over the oil shell permeability and limited access to the droplet interior present serious drawbacks. Here, a straightforward strategy for studying bacteria‐encapsulating double emulsion‐templated giant unilamellar vesicles (GUVs) is introduced. This microfluidic approach serves to simultaneously load bacteria inside synthetic GUVs and to permeabilize their membrane with the pore‐forming peptide melittin. This enables antibiotic delivery or the influx of fresh medium into the GUV lumen for highly parallel cultivation and antimicrobial efficacy testing. Polymer‐based GUVs proved to be efficient culture and analysis microvessels, as microfluidics allow easy selection and encapsulation of bacteria and rapid modification of culture conditions for antibiotic development. Further, a method for in situ profiling of biofilms within GUVs for high‐throughput screening is demonstrated. Conceivably, synthetic GUVs equipped with biopores can serve as a foundation for the high‐throughput screening of bacterial colony interactions during biofilm formation and for investigating the effect of antibiotics on biofilms.

## Graphical abstract

Permeability‐engineered giant unilamellar vesicles (GUVs) are presented as a method for the highly parallel cultivation and screening of microorganisms. This method enables high‐throughput screening of bacterial cultures and provides diverse cultivation environments. The long‐term stability of the GUVs and their suitability for various analytical methods make this strategy a basis for more advanced high‐throughput models of biofilms, organelles, and cells.

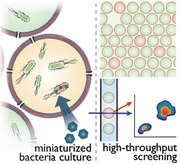

## 1 Introduction

Antibiotic resistance poses a major threat to modern healthcare. Thus, it is critical to understand the underlying mechanisms of antibiotic resistance in order to develop novel therapeutics that circumvent resistance.^[^ ^1^, ^2^, ^3^ ^]^ In some cultures of genetically identical bacteria, subpopulations emerge that divide labor and exchange metabolites to reduce the colony's sensitivity to stress and to increase growth efficiency and robustness.^[^ ^4^ ^]^ Population heterogeneity is an important phenomenon that allows bacteria to survive antibiotic treatment through the development of persister cells that tolerate antibiotics and subsequently give rise to new, resistant bacterial populations.^[^ ^5^ ^]^ To gain a better understanding of these persister effects, single‐cell or single‐colony models are required, demanding the use of high‐throughput methods to screen colony viability.

Typical formats for culturing bacteria include broth cultures, nutrient agar plates,^[^ ^6^, ^7^, ^8^ ^]^ and 96‐well plates.^[^ ^9^, ^10^ ^]^ While computational methods and faster processing have increased the throughput of these procedures, screening a wide range of antimicrobials is still a laborious process. Microfluidic devices allow the creation of microenvironments for long‐term cultivation of bacteria and subsequent analyte screening but are restricted with regard to reusability and device fabrication. Besides, analysis is often limited to qualitative methods.^[^ ^11^, ^12^, ^13^, ^14^ ^]^ An important advance in the highly parallelized cultivation and screening of single cells and microbial colonies is the use of double emulsions. Bacteria can be encapsulated in water/oil/water double emulsions, where – given appropriate conditions – they can grow and be further analyzed using high‐throughput methods, such as flow cytometry.^[^ ^14^, ^15^, ^16^, ^17^ ^]^ However, the limited control over the permeability of the droplet oil shell and the restricted access to the interior of the droplet often limit the applicability of double emulsion‐based culturing systems. A more readily modifiable and controllable alternative to thin‐shelled water/oil/water double emulsions for the biomimetic encapsulation of aqueous phases are giant unilamellar vesicles (GUVs), that is, vesicles ranging in size from 1 to 100 µm bounded by a unilamellar membrane of amphiphiles (e.g., lipids, copolymers or mixtures of thereof). GUV membranes are often prepared from phospholipids,^[^ ^18^, ^19^ ^]^ but amphiphilic block copolymers serve as an excellent alternative due to their greater chemical tunability and mechanical stability.^[^ ^20^, ^21^ ^]^ Polymer GUVs are often inherently impermeable to small molecules,^[^ ^22^, ^23^ ^]^ but selective permeability and diffusion into the cavity can be achieved by introducing pores, transporters, or ionophores into the membrane without compromising vesicle architecture. Polymeric GUVs are usually prepared by bulk methods such as electroformation and film rehydration.^[^ ^24^, ^25^ ^]^ More recently, double emulsion microfluidics have taken the stage in GUV formation, enabling the rapid production of vesicles with low size dispersity and very high encapsulation efficiency.^[^ ^19^, ^23^, ^26^ ^]^ Polymer GUVs are suitable for advanced screening methods commonly used for cells, for example, flow cytometry,^[^ ^25^, ^27^, ^28^ ^]^ or droplet assays,^[^ ^29^, ^30^ ^]^ and are often used as catalytic microcompartments,^[^ ^23^, ^25^ ^]^ or as models for artificial cells.^[^ ^31^, ^32^, ^33^ ^]^ However, their application as biohybrid culture compartments to study bacterial growth in confinement is still limited, mostly because of inefficient production in bulk and restricted access to the inner cavity after production.^[^ ^34^, ^35^, ^36^, ^37^ ^]^ More importantly, polymeric GUVs have so far not been used for testing antibiotics.

**Figure 1:**
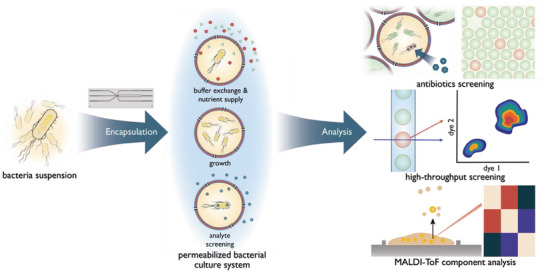
Workflow of bacteria encapsulation in double emulsion templated GUVs; bacteria are grown in a medium and injected into the Si‐glass microfluidic chip via the inner aqueous phase, yielding double emulsions that mature into GUVs by solvent evaporation. Pores mediate the supply of encapsulated bacteria with fresh buffer and nutrients for growth and enable entry of test compounds targeting growth. GUVs can be analyzed using high‐throughput methods, such as flow cytometry. Matrix‐assisted laser desorption/ionization time‐of‐flight mass spectroscopy (MALDI‐ToF MS) allows for the analysis of the inner components of the GUVs.

Here, we introduce a strategy to simultaneously culture and analyze bacteria inside biopore‐permeabilized polymeric GUVs that were produced by optimized double emulsion microfluidics. Specifically, we tailored polymeric GUVs to serve as microincubators that lend themselves to the analysis of bacterial viability and antibiotic resistance by flow cytometry (**Figure** **1**). We chose the diblock copolymer polydimethylsiloxane‐*block*‐poly(2‐methyl‐2‐oxazoline) (PDMS‐*b*‐PMOXA) to generate the GUVs because this amphiphilic copolymer has been shown to self‐assemble into stable vesicles with a membrane thickness that can be controlled by changing the block size and composition.^[^ ^38^, ^39^ ^]^ An advantage of using diblock instead of triblock copolymers is the formation of membranes with a bilayer morphology without entanglements, that will allow an easier combination with biomolecules.^[^ ^40^ ^]^ In addition to self‐assembly methods from bulk, PDMS‐*b*‐PMOXA and corresponding triblock copolymers have been recently used to produce stable GUVs by microfluidic techniques. While oxygen can diffuse across PDMS‐based block copolymer membranes, water, small molecules, or nutrients are unable to pass.^[^ ^22^, ^23^, ^24^, ^41^ ^]^ Therefore, the use of permeabilizing approaches, including the insertion of biopores and channel porins is necessary to allow flux through the membrane.^[^ ^23^, ^42^, ^43^ ^]^ Here, we used melittin, an amphiphilic peptide that creates pores in membranes by changing its conformation from a random coil to an α‐helix when inside an amphiphilic bilayer.^[^ ^44^ ^]^ Melittin pores afford not only nutrient supply to the GUV interior, but they can be used to study the effect of externally added compounds on bacterial growth. A delicate balance at the molecular level is required to insert the biopores without affecting the integrity of the GUVs and to simultaneously provide conditions for in situ bacterial growth upon encapsulation inside the lumen of GUVs. To obtain stable GUVs under conditions that have no adverse effect on bacteria, we optimized several parameters, including viscosity, osmolarity, and buffer composition, and their influence on bacterial growth. By observing individual GUVs encapsulating bacteria using confocal laser scanning microscopy (CLSM), we studied the growth of bacteria in GUVs, and by screening at the population level using high‐throughput techniques such as flow cytometry, we studied the response of bacteria to external conditions. Through pores in the membrane, testing of a multitude of analytes is possible, as we demonstrate for multiple antibiotics. We further analyzed the internal composition of GUVs using matrix‐assisted laser desorption/ionization time‐of‐flight mass spectroscopy (MALDI‐ToF‐MS) to confirm the presence of biofilms within GUVs. Using flow cytometry, we demonstrate high‐throughput screening of antibiotic resistance in bacterial colonies to improve the understanding of persister effects in bacteria.

## 2 Results and Discussion

### 2.1 Production of Double Emulsion Templated GUVs

**Figure 2:**
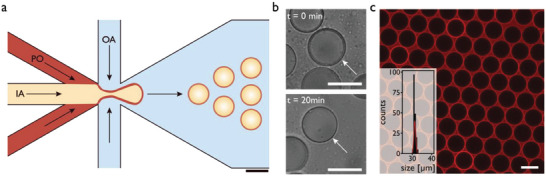
a) Schematic representation of the production of double emulsions using a microfluidic six‐way junction. Scale bar corresponds to 50 µm. b) Transmission micrograph of a single vesicle (arrow) dewetting from a double emulsion to a GUV over 20 min. c) Fluorescent micrograph of GUVs with the PDMS‐*b*‐PMOXA membrane stained with BODIPY 630/650. Inset represents a size histogram and regressed normal distribution (red line) of 187 measured GUVs with a diameter of 31.3 ± 0.5 µm. Scale bars, 30 µm.

Double emulsion GUV templates were prepared in a one‐step microfluidic droplet formation process using a six‐way junction (**Figure** **2**a) in a silicon‐glass microfluidic chip (Figure S1, Supporting Information). For the formation, an inner aqueous (IA) phase and an enclosing polymer organic (PO) phase, containing the PDMS_25_‐*b*‐PMOXA_10_ block‐copolymer, were co‐flowed, while an outer aqueous phase (OA) was cross‐flowed to break up the fluids into monodisperse double‐emulsion droplets. These were generated at a rate of ≈ 0.3 kHz (Figure S2, Supporting Information). The composition of the IA and OA phases was optimized in terms of the physicochemical properties of the fluids, such as osmolarity and viscosity, and to create a microenvironment conducive to bacterial survival and growth while ensuring the stability of the produced vesicles. We included high molecular weight polyethylene glycol (PEG, average M_n_ 35 000) rather than short‐chain PEG to increase the viscosity of the medium without the strong osmotic effect of the latter.^[^ ^23^, ^45^ ^]^ To increase vesicle stability, we also replaced the polyvinyl alcohol (PVA) commonly employed in the OA with long‐chain PEG supplemented with 0.1% of the surfactant Pluronic F‐68 and 0/100 mм sodium chloride (Figure S3, Supporting Information).^[^ ^23^, ^26^ ^]^ The influence of the osmolarity difference between the outer aqueous and the inner aqueous phase was examined and high stability was observed in most combinations for at least seven days at room temperature and 37 °C (Figure S4, Supporting Information). These adaptations allowed us to generate double emulsion templates under conditions that imitate standard bacterial culture conditions that is key to not affecting bacteria in the course of loading them inside GUVs. For GUVs to from the initial double emulsions, the organic solvent, a 3:2 mixture of hexane:chloroform used to dissolve the amphiphilic block copolymer, had to be removed. This was achieved by simply exposing the double emulsion droplets to air where evaporation of the volatile organic solvent turned them into stable GUVs.

The shrinking of the organic phase occurred within a few minutes as revealed by time‐lapse optical microscopy of freshly prepared double emulsions in an airtight chamber (Figure 2b). After solvent evaporation, a residual polymer pocket was often observed on the GUV surface that could not be completely removed (Figure S5, Supporting Information). For size determination, GUV membranes were stained with the lipophilic dye BODIPY 630/650 and imaged by confocal laser scanning microscopy (CLSM) (Figure 2c). The CLSM micrographs were analyzed by a Circle Hough Transform algorithm (Figure S6, Supporting Information), yielding a mean GUV diameter of 31.3 ± 0.5 µm (± 1.6%).

The double‐emulsion templated approach discussed here initially provides highly uniform GUVs. Uniformity is of great importance when comparing biofilm growth kinetics such as to avoid size‐dependent subpopulations within an individual sample. This is even more important for high‐throughput screening methods based on individual microcultures. Using cryo‐transmission electron microscopy, the thickness of PDMS_25_‐*b*‐PMOXA_10_ membranes was previously determined to be 12.0 ± 0.8 nm.^[^ ^46^ ^]^

### 2.2 Encapsulation of Bacteria in GUVs

The encapsulation of living bacteria in large numbers of GUVs requires a fast and simple workflow with high reproducibility. In double emulsion microfluidics, bacteria can be directly loaded via the IA phase, which minimizes handling time. For this purpose, bacteria were grown as suspension cultures until they reached the exponential growth phase (optical density at 600 nm (OD_600_) = 0.2–0.8), washed with fresh medium, resuspended at different concentrations, and then applied to the microfluidic device under gentle stirring to prevent bacteria from sedimenting without affecting cell viability (Figure S7, Supporting Information). Double emulsions that had formed around the bacteria‐containing inner aqueous phase were then collected in an open tube where they matured into GUVs under air exposure over a period of one hour.

**Figure 3:**
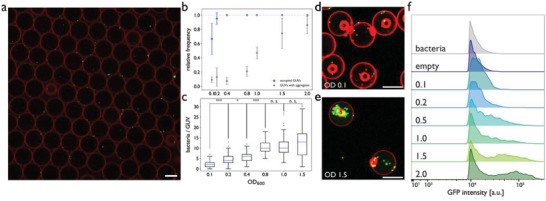
a) Fluorescence micrograph of GFP‐expressing *E. coli* (green) inside GUVs stained with BODIPY 630/650. b) GUV occupancy and fraction of GUVs with aggregates depending on the concentration of the bacterial suspension added to the double emulsion microfluidics, as determined by fluorescence microscopy. Data represented as mean ± SD. c) Determination of bacteria per GUV based on fluorescence microscopy image analysis (n = 30–60 GUVs per condition). One‐way ANOVA was used for comparison: *p* > 0.05 (n.s.), *p* < 0.05 (*), *p* < 0.005 (**), *p* < 0.0005 (***), and n.s. = not significant, Tukey's post hoc test. d+e) Representative fluorescence micrographs of GFP‐expressing *E. coli* (green) encapsulated within PDMS‐*b*‐PMOXA GUVs stained with BODIPY 630/650 (red) at input OD_600_ of d) 0.1 (≈ 8.0 × 10^7^ cells mL^−1^) and e) 1.5 (≈ 1.2 × 10^9^ cells mL^−1^). f) Flow cytometric analysis of GUVs with encapsulated GFP‐expressing *E. coli* depending on input bacterial concentration. Scale bars, 30 µm.

Close control over the encapsulation efficiency and number of bacteria loaded per GUV is essential for studying their growth behavior in individual GUVs. To distinguish GUVs harboring bacteria from empty ones and thus determine encapsulation efficiency, we used GFP‐expressing *Escherichia coli* (*E. coli*) that were diluted to a wide range of different concentrations. Initial concentrations of bacteria suspension cultures in the range of OD_600_ = 0.1 – 2.0 (≈ 8.0 × 10^7^ – 1.6 × 10^9^ cells mL^−1^) were used for encapsulation. The membranes of the resulting GUVs were stained with the hydrophobic dye BODIPY 630/650 (**Figure** **3**a, red) and GUVs were imaged by CLSM. Micrographs were used to analyze the number of bacteria‐containing GUVs in at least 30 GUVs per condition from three independent loading experiments. At low bacteria concentrations (OD_600_ < 0.8 ≈ 8.0 × 10^8^ cells mL^−1^), 5–10% of the GUVs were empty whereas at higher concentrations (OD_600_ ≥ 1.0) each GUV was loaded with GFP‐bacteria (i.e., 0% empty GUVs) (Figure 3b). We next determined the number of bacteria encapsulated per GUV (Figure 3c). At concentrations ranging from OD_600_ ≃ 0.1 – 0.4, the encapsulation efficiency consistently increased (2.4–5.7 ± 1.3–2.3 bacteria GUV^−1^) and was in agreement with theoretical loading values calculated from the GUV volume and input bacterial concentration (Figure S8, Supporting Information). At loading concentrations OD_600_ > 0.4, the number of bacteria per GUV was significantly higher but variability noticeably increased (Figure S23 and Table S8, Supporting Information), suggesting that at high input concentrations, the number of encapsulated bacteria could not be well controlled. It is likely that a higher fraction of bacterial aggregates in the loaded suspension (Figure 3b,e) interfered with an accurate count of encapsulated bacteria.^[^ ^47^ ^]^ In order to maintain the highest control over the encapsulation efficiency of bacteria, the bacterial concentration suitable for loading was determined to be between 0.2 < OD_600_ < 0.8.

To evaluate the presence of bacteria in individual GUVs at high‐throughput, we employed flow cytometry. To determine whether GFP‐expressing *E. coli* inside GUVs can be evaluated based on their green fluorescence, the detected events were gated based on their forward scattered signals (FSC‐A vs FSC‐H, typical for large particle analysis) to eliminate aggregated GUVs or debris (Figure S9, Supporting Information). Fluorescence intensity was then processed for the gated populations and analysis revealed increasing green‐fluorescence with increasing loading, with ≈tenfold higher fluorescence intensities associated with GUVs loaded with the highest number of bacteria (Figure 3f) when compared to the empty GUVs. Consistent with the data obtained by CLSM, the clear shift to higher fluorescence intensities at OD_600_ > 0.5 pointed to aggregates, indicating that the input concentration of the bacteria should be between OD_600_ values of 0.2 and 0.5 to minimize loading aggregates. When comparing the two methods for quantifying bacteria in GUVs, a minimum input concentration of OD_600_ = 0.4 was found to be ideal for sufficient bacterial loading that can also be detected by flow cytometry.

### 2.3 Influence of Medium on Growth of GUV‐Confined Bacteria

**Figure 4:**
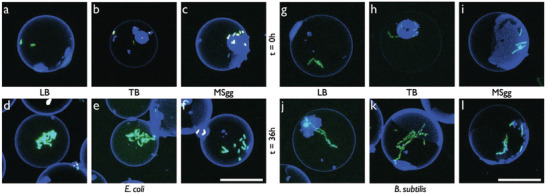
Growth behavior of wildtype *E. coli* and *B. subtilis* in different media. Bacteria were encapsulated at a starting OD_600_ = 0.4. Representative CLSM images of a‐f) SYTO 9‐stained *E. coli* (green) in GUVs (blue) in LB (a+d), TB (b+e) and MSgg (c+f) medium before a–c) and after d—f) incubation at 37 °C for 36 h. g–l) SYTO 9‐stained *B. subtilis* (green) in LB (g+j), TB (h+k), and MSgg (i+l) medium before g–i) and after j–l) incubation at 37 °C for 36 h. Scale bars, 30 µm.

The medium in which bacteria are cultured significantly impacts their growth. To assess the applicability of GUV‐encapsulated bacteria to high‐throughput screening, we tested the impact of different standard bacterial growth media, that is, Lysogeny Broth (LB), Terrific Broth (TB) and Minimal Salts glutamate glycerol (MSgg) on encapsulated *E. coli* and *Bacillus subtilis* (*B. subtilis*). *B. subtilis* is known to readily form biofilms – surface‐associated multicellular communities that are resistant to various environmental stresses such as extreme pH, high salinity, and antibiotics.^[^ ^48^, ^49^, ^50^ ^]^ In nutrient‐rich media (LB/TB), *E. coli* were found to swim freely inside freshly produced GUVs (**Figure** **4**a,b) and multiplied over 36 h at 37 °C (Figure 4d,e). With an estimated doubling time (DT) of 10 h, the DT in GUVs is much higher than that of planktonic *E. coli* grown under aerobic, nutrient‐rich conditions at 37 °C in shaking incubators (≈ 3 h, Figure S10, Supporting Information) but more closely matches that of *E. coli* in the wild, which has been estimated at ≈ 15 h.^[^ ^51^ ^]^ A similar trend was observed for *B. subtilis* grown in LB or TB (Figure 4g–k). Both strains showed minimal growth when cultured in biofilm‐promoting MSgg (Figure 4f,l). Clearly, for compound screening, growth media must be adapted to the respective bacterial strain to provide adequate growth conditions such as nutrients and buffers.

### 2.4 Engineering the Permeability of GUVs

**Figure 5:**
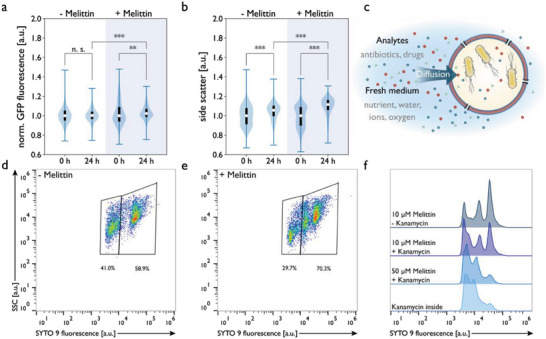
Flow cytometric analysis of GFP‐expressing *E. coli* encapsulated at OD_600_ = 0.4 in GUVs with and without melittin permeabilization (n = 3000–6000 GUVs) and incubated overnight at 37 °C. Violin plots of a) GFP fluorescence and b) side scattering (SSC) evaluated per GUV. Two‐way ANOVA was used for comparison: p > 0.05 (n.s.), *p* < 0.05 (*), *p* < 0.005 (**), *p* < 0.0005 (***), and n.s. = not significant, Tukey's post hoc test. c) Schematic of the bacteria‐encapsulating vesicles. Bacteria are encapsulated within polymeric GUVs and through pores, analytes, and fresh medium can diffuse into the lumen of the GUV. Flow cytometry analysis of *B. subtilis* growth inside GUVs after 72 h d) without melittin pores and e) with 10 µм melittin added to the outer aqueous phase (n = 5300–8000 GUVs). f) Histogram of SYTO 9 fluorescence from flow cytometry of *B. subtilis* GUVs permeabilized with 10 or 50 µм melittin exposed to 50 µg mL^−1^ kanamycin from the outside after production and GUVs containing *B. subtilis* with 50 µg mL^−1^ kanamycin inside (n = 5300–8000 GUVs). Significance levels: *p* > 0.05 (n.s.), *p* < 0.05 (*), *p* < 0.005 (**), and *p* < 0.0005 (***).

Rendering the polymeric membrane permeable to nutrients and test compounds is essential for the application of GUVs for bacterial growth and testing (**Figure** **5**c). While PDMS‐*b*‐PMOXA vesicles have shown permeability to O_2_, ions and hydrophilic small molecules are not able to pass the polymer membrane (Figure S11, Supporting Information). To enable communication between GUV interior and exterior, we permeabilized the GUV membranes by insertion of melittin, the main component of honey bee venom. Melittin has been inserted in polymer membranes of triblock copolymers by addition at various steps of the vesicle formation process (in the bulk polymer film, added to the rehydration buffer, or added to already formed polymersome dispersions).^[^ ^52^ ^]^ However, the microfluidic approach represents a completely different strategy for biopore insertion and is therefore providing other conditions for the insertion of melittin and its assembly into functional pores. Specifically, we employ melittin as a component during the double‐emulsion formation, resulting in melittin‐associated double emulsions that then through de‐wetting evolve into melittin‐functionalized GUVs. Unlike other biopores known to permeabilize PDMS‐*b*‐PMOXA bilayers, for example, OmpF,^[^ ^53^ ^]^ melittin does not have a defined pore size but dynamically assembles pores within the membrane with openings up to 1.4 nm, corresponding to a molecular cutoff of ≈ 4 kDa.^[^ ^52^ ^]^

We assessed the permeabilization of GUVs using an enzymatic reaction. The enzyme β‐galactosidase was encapsulated in GUVs and the nonfluorescent substrate fluorescein di(beta‐D‐galactopyranoside (FDG, 656.6 Da) was added externally to show an increase in fluorescence intensity by the enzyme‐catalyzed generation of fluorescein in the lumen of GUVs (Figure S12, Supporting Information). We found that adding melittin to the outer aqueous phase during microfluidic double emulsion production was the most effective strategy for selective pore formation while still maintaining stability over seven days (Figure S13, Supporting Information). Besides, the addition of melittin is straightforward and will support the use of such permeabilized GUVs for high‐throughput screening approaches. This allowed us to avoid the biocidal effects of melittin on the encapsulated bacteria.^[^ ^54^ ^]^ The membrane permeabilization was further confirmed for relatively lipophilic cell‐permeant dyes, such as the nucleic acid stain SYTO 9 or the dead stain propidium iodide (PI) where no significant difference in bacterial staining could be observed between internal and external addition of dye (Figure S14, Supporting Information). Using quantitative fluorescence spectrometry, a local concentration of 2 × 10^7^ melittin monomers per GUV was determined, corresponding to up to 1.9 × 10^6^–7.9 × 10^6^ pores per GUV (Figure S21, Supporting Information).

The influx of fresh medium to promote growth was evaluated by adding GFP‐*E. coli* to the inner and 10 µм melittin to the outer aqueous phase for double emulsion formation. Ensuing GUVs were analyzed by flow cytometry fresh (0 h) and after 24 h incubation at 37 °C. Bacterial growth was assessed by comparing GFP fluorescence and side scatter (SSC) of permeabilized and non‐permeabilized GUVs. Both signals can be used as a measure of the number of bacteria per GUV, since GFP fluorescence and side scattering increase with increasing numbers of bacteria, the latter based on higher internal granularity of the GUVs. After 24 h at 37 °C, GUVs without melittin pores did not significantly change their GFP fluorescence, whereas melittin‐permeabilized GUVs (Figure 5a,b) showed a significant increase in both SSC and GFP fluorescence. While we did not observe a significant increase in GFP fluorescence in non‐permeabilized GUVs, the SSC signal increased significantly (Figure S24 and Tables S9 and S10, Supporting Information). This is possibly due to the strong scattering of GUVs and their surrounding buffers at different wavelengths, which could be reduced by a more extensive GUV washing protocol or by optimization of the flow cytometry measurements. Flow cytometry showed that bacteria initially grew in non‐permeabilized GUVs but stopped growing once the medium was spent (Figure 4a,d), in agreement with the data obtained by CLSM.

Bacterial growth was also analyzed in *B. subtilis*‐encapsulating GUVs, when grown for 72 h at 37 °C. SYTO 9 was added to the IA to stain bacteria, and the far‐red emitting dye Nile red was added to the polymer phase to render GUV membranes fluorescent. Non‐encapsulated bacteria and free dye were removed by washing bacteria‐GUV cultures prior to flow cytometry, and aggregated GUVs or debris were removed by gating in flow cytometry data analysis. Specifically, only Nile red‐positive GUVs were selected. In GUVs without melittin pores, we detected predominantly two populations with different SYTO 9 intensities (Figure 5d), corresponding to GUVs encapsulating too few bacteria to detect and GUVs with high fluorescence and encapsulating high numbers of bacteria. When 10 µм melittin was added to the OA, GUVs showed increased SYTO 9 fluorescence (Figure 5e) compared to non‐permeabilized GUVs (Figure 5d), indicating increased microbial growth when GUVs were permeabilized. Notably, part of the population with low fluorescence exhibited a high increase in fluorescence intensity, indicating that although the total number of GUVs containing growing bacteria did not increase significantly, permeabilization had a large effect on GUVs containing few bacteria, which then multiplied (Figure 5e).

A further advantage of membrane pores is that encapsulated bacteria can be exposed to externally added compounds to screen their effect on bacterial growth. To demonstrate the antibacterial activity of a test compound we added kanamycin, a model antibiotic that interferes with protein synthesis, to GUV microincubators. Specifically, we collected GUVs encapsulating bacteria in a buffer containing 50 µg mL^−1^ kanamycin, a concentration much higher than the minimal inhibitory concentration (MIC) determined for planktonic *B. subtilis* cultured in MSgg + 5% PEG_35 000_ (Figure S15 and Table S3, Supporting Information), and incubated at 37 °C for 72 h. In the presence of kanamycin, a decrease of fluorescence in melittin‐permeabilized *B. subtilis*‐GUVs was observed compared to corresponding GUVs cultured without kanamycin (Figure 5f). When GUVs were permeabilized at a low melittin concentration (10 µм), the decrease in fluorescence was predominantly observed in populations with high and medium fluorescence. At a higher melittin concentration (50 µм), the majority of GUVs exhibited low GFP fluorescence. Similarly, most bacteria in nonpermeabilized GUVs with the same concentration of kanamycin inside showed low fluorescence. This shows that the growth of encapsulated bacteria was largely inhibited by kanamycin at a concentration comparable to the maximum daily dose (15 mg kg^−1^ day^−1^ = 15 µg g^−1^ day^−1^) administered in the treatment of bacterial infections in humans.^[^ ^55^ ^]^ Inhibition of *B. subtilis* growth was not as efficient at lower concentrations of melittin (10 µм), however, these GUVs tended to be more stable than GUVs permeabilized with 50 µм melittin.

Taken together, these results suggest that GUVs have been successfully permeabilized with the pore‐forming peptide melittin and that kanamycin and nutrients can enter the GUVs through the assembled pores. Moreover, our GUV‐based screening approach is not limited to a single administration of the test compound but lends itself to repeated challenges, for example with an increasing dose of an antimicrobial compound.

### 2.5 Biopore‐Equipped GUVs for Testing Efficacy of Antibiotics Against Biofilms

**Figure 6:**
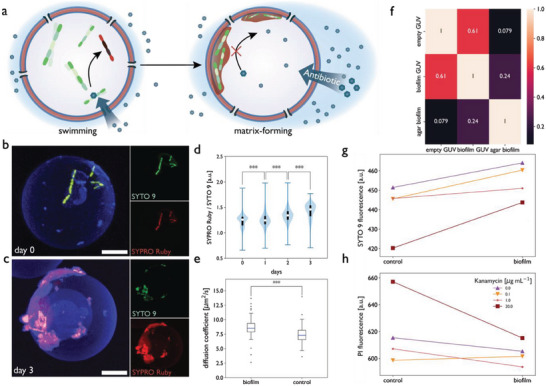
a) Schematic representation of bacterial biofilm colony growth within GUVs. *B. subtilis* are encapsulated in PDMS‐*b*‐PMOXA GUVs and grown for an extended duration to allow for matrix and biofilm formation. Sessile bacteria deposit matrix at the inner face of the vesicle membrane, leading to an increased antibiotic resistance. *B. subtilis*‐encapsulating GUVs b) before and c) after incubation in MSgg for 72 h at 30 °C. The nucleic acid stain SYTO 9 (green) and FilmTracer SYPRO Ruby Biofilm Matrix Stain (red) were used to stain the bacteria and biofilm matrix, respectively, and the GUV surface was functionalized with DBCO‐Cy5 (blue) that was linked to an azide end group on the polymer membrane. Scale bars, 10 µm. d) Violin plots showing the ratio of SYPRO Ruby biofilm matrix stain to SYTO 9 nucleic acid over time measured by flow cytometry (n = 2000–10000 GUVs). e) Measured polymer lateral membrane diffusion coefficients (D (µm^2^s^−1^)) for GUVs incubated for 72 h with and without *B. subtilis*. Measurements were performed and averaged for 10 GUVs per condition. f) Pearson correlation matrix of MALDI‐ToF‐MS comparing empty GUVs, GUVs containing biofilms, and a native biofilm grown on MSgg agar plates. Interaction plot showing the population mean of the g) SYTO 9 and h) PI fluorescence intensity of *B. subtilis*‐containing GUVs exposed to different kanamycin concentrations. Kanamycin is administered 2 h (control) or 48 h (biofilm) after production of GUVs and incubation at 30 °C (n = 1500–10000 GUVs). Two‐way ANOVA was used for comparison in d, g, and h: *p* > 0.05 (n.s.), *p* < 0.05 (*), *p* < 0.005 (**), *p* < 0.0005 (***), and n.s. = not significant, Tukey's post hoc test. Two‐tailed t‐test was used in e.

To reproduce biofilm formation in GUVs and apply them to high‐throughput screening applications, we studied the response of *B. subtilis* when grown in GUVs for several days under biofilm‐promoting conditions. The concomitant diffusion of nutrients and antibiotics through melittin pores is an essential prerequisite for testing the effect of antibiotics on bacterial growth, as exposing pre‐starved bacteria to antibiotics would distort the antibacterial potency of antibiotics. As indicated in **Figure** **6**a, encapsulated *B. subtilis* cultured in MSgg, a minimal medium that induces biofilm formation,^[^ ^53^ ^]^ accumulate at the inner liquid‐polymer interface of GUVs where they deposit a matrix that increases *B. subtilis* resistance against the bactericidal effects of antibiotics. To test this, GUVs containing *B. subtilis* were stained with the biofilm matrix dye SYPRO Ruby after culturing for three days in MSgg. CLSM images recorded on day 0 (Figure 6b) and day 3 (Figure 6c) reveal that while the number of bacteria increased only minimally, the intensity of the biofilm matrix staining (red) was much stronger on day 3. This observation was corroborated by flow cytometry, where the increase of the extracellular matrix relative to the number of bacteria was assessed by comparing the ratio of biofilm matrix (SYPRO Ruby biofilm matrix stain) to bacteria (SYTO 9 nucleic acid stain). Accordingly, the increase in the matrix‐to‐bacteria ratio (SYPRO Ruby/SYTO 9, Figure 6d; Figure S25 and Table S11, Supporting Information) indicated that the bacteria secreted matrix proteins and polysaccharides over time, which is consistent with the confocal images displaying stained matrix components (red) around the bacteria (Figure 6b,c). Moreover, the bacteria also appeared to develop an interaction with the polymer membrane. This was confirmed by colocalization CLSM imaging, correlating *B. subtilis* localization with the dye‐functionalized membrane (Figure S17, Supporting Information).

For fresh biofilm‐GUVs (day 0 after formation), a Mander's colocalization coefficient of 0.23 ± 0.08 was observed that increased to 0.58 ± 0.18 over the next three days, demonstrating significantly increased interaction of bacteria with the polymer membrane over time (Tukey's honest significant difference (HSD), *p* < 0.05 (Tables S5,S6, Supporting Information)).

The effects of bacterial matrix deposition within the polymer membrane were further investigated by measuring the membrane fluidity of GUVs using fluorescence recovery after photobleaching (FRAP). By inserting a fluorescent lipidic probe (0.1% 18:1 Liss Rhod PE) in the GUV membrane, we detected a significant (*p* < 0.05) increase in membrane fluidity compared to GUVs without bacteria (Figure 6e). We hypothesized that this effect might be caused by extracellular polymeric substances (EPS) such as lipids, proteins, polysaccharides, or nucleic acids secreted by bacteria that may be incorporated into or deposited onto the polymeric GUV membrane.^[^ ^56^ ^]^ We tested our hypothesis by incorporating the lipid POPC into the polymeric membrane during formation, and found that already the addition of 1% POPC significantly increases the membrane fluidity of empty GUVs to a level determined for biofilms (Figure S18, Supporting Information), consistent with the notion that the growth of *B. subtilis* in GUVs increased membrane fluidity by modifying the composition or structure of the membrane.

The development of biofilms inside GUVs was further characterized by MALDI‐ToF mass spectroscopy of the content of the polymer vesicles. We applied a protocol of repeated washing of GUVs that allowed the deposition of the vesicles on target plates while removing noise‐inducing components of the OA. This allowed us to measure cargo loaded in GUVs, as exemplified by melittin that was encapsulated inside GUVs (Figure S19, Supporting Information). MALDI‐ToF mass spectra of GUVs with and without growing bacteria were recorded and compared to spectra of a native biofilm conventionally grown on agar plates in a minimal medium (Figure S20, Supporting Information). Using the Pearson correlation coefficient, the spectra were compared (Figure 6f), and a threefold higher correlation was found between native biofilms and *B. subtilis*‐primed GUVs than between native biofilms and empty GUVs. Although this suggests the presence of *B. subtilis* biofilm proteins in GUVs, complete retrieval of the spectra of biofilm‐associated proteins was not feasible due to the overlap of polymer‐specific peaks and biofilm proteins. While further in‐depth optimization would be required to unambiguously assign the spectra, the MALDI‐ToF data suggest that GUVs are suitable for establishing biofilm compartments for high‐throughput screening. In addition, our results indicate that high‐throughput mass spectroscopic screening will be possible in the future by combining MALDI‐ToF‐mass spectroscopy and fluorescence‐assisted cell sorting (FACS)

Because kanamycin is known to reduce biofilm mass in pre‐existing *B. subtilis* biofilms,^[^ ^54^ ^]^ we tested its efficacy in GUVs containing *B. subtilis* biofilms. Melittin‐permeabilized GUVs were cultured in MSgg for 48 h to allow for biofilm formation and subsequently exposed to kanamycin at different concentrations. Alternatively, GUVs were treated with different kanamycin concentrations immediately after GUV formation, where biofilm formation had not yet occurred. In order to show that melittin permeabilization of the GUV membranes persisted over the incubation period, we determined pore localization at different time points in GUVs using FITC‐functionalized melittin and observed that neither long‐term incubation at 37 °C nor the presence of bacteria or biofilms decreased the affinity of melittin to the membrane (Figure S21, Supporting Information). In both cases, GUVs were stained with propidium iodide (PI) to identify dead cells after 24 h of kanamycin treatment and analyzed by flow cytometry. Figure 6g shows the side scatter and PI fluorescence of GUVs containing *B. subtilis* for four different concentrations of kanamycin that were added immediately after GUV formation (purple, control) or after 48 h preculture at 30 °C (red, biofilm), assuming that *B. subtilis* formed a biofilm during this time (Figure S22, Supporting Information). At kanamycin concentrations below the MIC (< 1.3 µg mL^−1^) little bactericidal effect is observed independent of whether the GUVs were precultured or not. At time points prior to biofilm formation, *B. subtilis* GUVs exposed to 20 µg mL^−1^ kanamycin immediately after GUV formation (≈ 15 × MIC, Figure 6g) showed a twofold increase in PI stain whereas this concentration had virtually no effect if biofilm formation was initiated during preculture. These results show that in the absence of biofilm formation, *B. subtilis* is more susceptible to kanamycin and that biofilm structures increase the bacteria's resistance to kanamycin (Figure S26 and Tables S12 and S13, Supporting Information).^[^ ^57^ ^]^

**Figure 7:**
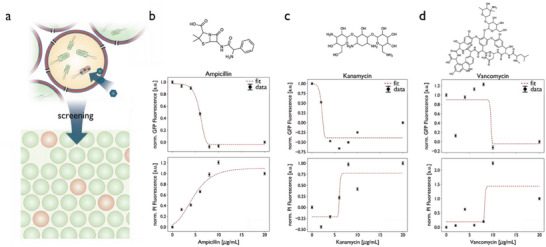
a) Schematic illustration of antibiotics testing in bacteria‐loaded GUVs. GFP (top row) and PI (bottom row) fluorescence profiles of bacteria‐GUVs exposed to different concentrations of b) ampicillin, c) kanamycin, and d) vancomycin. Observed fluorescence was fitted to a four‐parametric logistic regression (red). Data are normalized and mean and 95% confidence interval is shown (black) (n = 3). One‐way ANOVA was used for comparison in d, g, and h: *p* > 0.05 (n.s.), *p* < 0.05 (*), *p* < 0.005 (**), *p* < 0.0005 (***), and n.s. = not significant, Tukey's post hoc test.

To further explore the applicability of the bacteria‐GUVs for high‐throughput screening, a selection of antibiotics at a wide range of concentrations (1‐20 µg mL^−1^) were tested for their effect on the GUV populations (**Figure** **7**a). Ampicillin, kanamycin, and vancomycin, which represent a broad range of molecular weights (349–1449 Da), were tested at concentrations above and below their respective MICs. Melittin‐permeabilized GUVs containing GFP‐expressing *E. coli* were prepared and incubated in antibiotic‐containing OA supplemented with PI for 4 h at 37 °C. Permeabilization with melittin pores facilitates the influx of fresh medium, which promotes bacterial growth and enhances GFP fluorescence by increasing GFP‐expressing bacteria. At the same time, the influx of antibiotics is allowed, which inhibits bacterial growth and kills the bacteria, increasing the fluorescence of the dead stain PI. After washing, the GUVs were analyzed using flow cytometry to determine the bacteria's growth and the fraction of dead bacteria inside the GUVs (Figure 7a). A clear trend of increasing PI‐intensity and decreasing GFP‐intensity is shown in response to increasing antibiotic concentrations (Figure 7b–d), reflecting the bactericidal effects of the antibiotics. The determination of the MIC upon fitting of the responses with a Gompertz function was consistent in both the GFP and PI signals (1.1–3.4 µg mL^−1^ depending on an antibiotic) and was further confirmed by a conventional MIC assay (Figure S16 and Table S4, Supporting Information).^[^ ^58^ ^]^ Furthermore, significant differences of GFP and PI fluorescence intensities were identified between concentrations of antibiotics in the range of the MIC (Tukey's honest significant difference (HSD), *p* < 0.05, see Figure S27 and Tables S14–S19, Supporting Information). These results indicate the suitability of bacteria‐encapsulation polymeric GUVs for high‐throughput screening assays, that is, for the determination of the MIC of bacterial colonies in accordance with conventional 96‐well assays. This method allows bacteria and bacterial colonies to be encapsulated in permeabilized GUVs and grown to a desired density before exposure to antibiotics, enabling high‐throughput antibiotic screening of bacterial cultures.

## 3 Conclusion

We have introduced giant unilamellar vesicles equipped with biopores generated by double emulsion microfluidics as versatile, micrometer‐sized compartments for growing bacteria as individual cultures. The present approach of using GUVs produced by microfluidics for culturing bacteria differs significantly from other means of culturing such as suspension cultures or inside double emulsions. The unprecedented advantages of the microfluidic production of bacteria‐loaded GUVs are its rapidity, high reproducibility, and the ability to provide GUVs well‐defined in size and composition. These features are critical for comparative studies and a prerequisite for both high‐throughput production and screening of bacterial microenvironments. The unique properties, adaptability, and stability of polymeric membranes allow the system to be tailored to a variety of experimental requirements, such as different pores or changes in the environment. By taking advantage of microfluidics, the bacterial microenvironment can be precisely modified by the addition of corresponding media or antibiotics without the need for specialized instrumentation or restriction to specific classes of molecules. Access to the GUV interior was provided by permeabilizing the polymer membrane with the pore‐forming peptide melittin. The pores proved suitable for the efficient antibiotic delivery of both nutrients and antibiotics. The GUVs furthermore proved suitable for the production of single‐colony biofilms, which formed under appropriate nutrient conditions without disrupting the GUVs. The enclosed biofilms showed an increased resistance to kanamycin. As biofilms are highly relevant for the emergence of antibiotic resistance, the possibility of analyzing their composition presents an important advance in compound screening. The all‐microfluidic production of the initial double emulsions provides high modularity, permitting, for example, the swapping of source bacterial culture “on the fly”. We are convinced that the strategy for producing bacterial colonies within GUVs has major advantages over currently used microfluidic cultivation methods for bacteria and biofilms, such as control over membrane properties, external access to the internal cavity, and versatile choice of medium. The application of microfluidic technologies to produce large numbers of individual bacterial cultures with controlled microenvironments lays the ground for faster and more efficient screening methods with important applications including antibiotic testing. In combination with fluorescence‐assisted cell sorting (FACS), persister colonies could be isolated and resistance mechanisms better understood, for example, by applying single‐cell sequencing.

## 4 Experimental Section

### Materials, Chemicals, and Strains

Polyethylene glycol (PEG, Mn = 35 000), polyvinyl alcohol (PVA, MW 13 000–23 000, 87–89% hydrolyzed), chloroform (99%), anhydrous hexane (95%), fluorescein di‐(β‐D‐galactopyranoside) (FGD), sucrose, sodium chloride, kanamycin sulfate (from Streptomyces kanamyceticus), α‐cyano‐4‐hydroxycinnamic acid, super‐DHB, melittin (from honey bee venom, ≥ 85% by HPLC), TWEEN20, trifluoroacetic acid (TFA), potassium phosphate mono‐ and dibasic, 3‐(N‐morpholino)propanesulfonic acid (MOPS), CaCl_2_, MnCl_2_, FeCl_3_, ZnCl_2_, thiamine, glycerol, glutamate, tryptophan, and phenylalanine were obtained from Sigma–Aldrich. Pluronic F‐68 Non‐ionic Surfactant (100X) was obtained from Gibco. MgCl_2_ was obtained from Fluka. H_2_SO_4_ was purchased from VWR. BODIPY 630/650, SYTO 9 Green Fluorescent Nucleic Acid Stain, and FilmTracer SYPRO Ruby Biofilm Matrix Stain were obtained from Thermo Scientific Inc. Cy5‐labeled melittin was purchased from Discovery Peptides. Bacterial tryptone, yeast extract, and agarwere purchased from Becton Dickinson. 18:1 Liss Rhod PE was obtained from Avanti Polar Lipids. Aquapel was obtained from PGW Auto Glass. All chemicals were used as received unless stated otherwise.

LB medium was composed of 1% tryptone, 0.5% yeast extract, and 1% w v^−1^ NaCl. MSgg medium was composed of 5 mм potassium phosphate (pH = 7), 100 mм MOPS (pH = 7), 2 mм MgCl_2_, 700 µм CaCl_2_, 50 µм MnCl_2_, 50 µм FeCl_3_, 1 µм ZnCl_2_, 2 µм thiamine, 0.5% glycerol, 0.5% glutamate, 50 µg mL^−1^ tryptophan, and 50 µg mL^−1^ phenylalanine.^[^ ^59^ ^]^ Milli‐Q water (resistivity of ≥ 18 MΩ cm‐1 at 25 °C) was obtained from a Millipore Sigma Milli‐Q Direct Water Purification System. *Bacillus subtilis* (Ehrenberg) Cohn ATCC 6051 (*B. subtilis*) was obtained from ATCC. JM101 *Escherichia coli* K12 (*E. coli*) was obtained from Novagen. *E. coli* ASC662 with a fusion lacZ‐gfp in native lac operon was obtained from the van Nimwegen lab.^[^ ^60^ ^]^

### Synthesis and Characterization of PDMS_25_‐b‐PMOXA_10_ and PDMS_27_‐b‐PMOXA_8_‐PEG_3_‐N_3_

The syntheses and characterization of amphiphilic diblock copolymers poly(dimethylsiloxane)_25_‐*b*‐poly(2‐methyl‐2‐oxazoline)_10_ (PDMS_25_‐*b*‐PMOXA_10_) and azide‐functionalized PDMS_27_‐*b*‐PMOXA_8_‐PEG_3_‐N_3_ were described previously.^[^ ^61^ ^]^ A membrane thickness of 12 ± 0.8 nm for self‐assembled PDMS_25_‐*b*‐PMOXA_10_ membranes was previously determined from cryogenic transmission electron micrographs.^[^ ^46^ ^]^

### Microfluidic Device Fabrication

A microfluidic silicon‐glass chip with a six‐way junction was used (Figure S1, Supporting Information), as described previously.^[^ ^23^ ^]^ Silicon‐glass microfluidic chips were fabricated in the Binnig and Rohrer Nanotechnology Center at IBM Research Europe – Zurich by deep reactive ion etching of microfluidic structures into Silicon wafers, followed by anodic bonding of Borofloat 33 (BF33) glass covers equipped with previously machined through‐holes as in‐ and outlet ports.

### Microfluidic Setup

All liquids used for chip coating were passed through suitable hydrophilic or hydrophobic syringe filters (pore size 0.2 or 0.45 µm) before use. A three‐module precision syringe pump (Cetoni neMESYS low pressure) was used for chip coating and to inject the fluid phases into the microfluidic chip. Hamilton gastight glass syringes (0.5, 1, and 5 mL) with PTFE luer lock adapters and fluorinated ethylene propylene (FEP) tubing (BGB Analytik) with an inner diameter of 0.25 mm (1/16″) were used for liquid handling. When bacteria were used, a syringe stirrer (Cetoni neMIX syringe stirrer with stirrer syringe) was used to prevent sedimentation of bacteria in the syringe. Stirring was set to level three.

### Microfluidic Device Coating

The microfluidic chips were rinsed with ultrapure Milli‐Q water and dried with N_2_ prior to surface activation in oxygen plasma for 15 min at ≈ 350 mTorr. For hydrophobic coating of the inner aqueous (IA) and polymer organic (PO) inlet channels of the six‐way junction, Aquapel (PGW Auto Glass) was injected through the inner aq. channel. A counterflow of N_2_ was used to confine the flow to only one side of the six‐way junction, pushing out Aquapel through the PO phase channels. The N_2_ gas flow was set to 1 mL min^−1^ with a gas mass flow controller (Bronkhorst EL‐FLOW Select). Aquapel was injected at 3–6 µL min^−1^ for 7 min, followed by thorough rinsing of the microfluidic chips with isopropanol, ultrapure water, 5% PVA (Mw 13000‐23000, 87–89% hydrolyzed) in ultrapure water, and ultrapure water, injected successively through the outlet channel. For chip regeneration, the chips were cleaned using ultrapure water and piranha solution (conc. H_2_SO_4_: 30% aq. H_2_O_2_ ≈ 7:3), followed by thorough rinsing with ultrapure water.

### Double Emulsion Fabrication and Dewetting

For the inner aqueous phase, the medium was supplemented with 5% w v^−1^ PEG_35 000_. The diblock copolymers were dissolved at 4 mg mL^−1^ in a 3:2 v v^−1^ mix of hexane and chloroform. For the outer aqueous phase, 5% w v^−1^ PVA_13 000−23 000_ (87–89% hydrolyzed) or 5% PEG_35 000_ and 0.1% Pluronic F‐68 was added to the medium. Further, 100 mм NaCl was added to increase the osmolarity of the outer aqueous phase. Osmolarity was measured with a freezing point osmometer (Gonotec Osmomat 3000). The different fluid phases were fed at flow rates of 1, 3, and 50 µL min^−1^ for the PO, IA, and OA phases, respectively. The double emulsions formed were collected for 10 min in an Eppendorf tube containing 300 µL OA phase, then exposed to air by pipetting them onto a microscope slide or leaving the tube open for several hours. Evaporation of the organic phase resulted in ≈ 10^6^ GUVs at a concentration of ≈ 10^6^ mL^−1^ (Figure S2, Supporting Information).

### Bacteria GUV Fabrication

Bacteria (GFP‐*E. coli*, *E. coli* JM101, and *B. subtilis*) from precultures were grown overnight in terrific broth (TB) or LB medium at 37 °C and 180 rpm. The next morning, the bacteria suspension was diluted to a concentration of OD_600_ = 0.05 and grown to OD_600_ = 0.4 – 0.6 (log phase). Bacteria were washed twice by centrifugation in assay medium for 3 min at 2700 × g at RT. Cultures were then diluted to the desired concentration and transferred to a glass syringe for microfluidic injection as the inner aqueous phase. *B. subtilis* and *E. coli* JM101 were additionally stained with 5 µм SYTO 9 green fluorescent nucleic acid stain.

### Confocal Microscopy and Image Analysis

For imaging by confocal laser scanning microscopy (CLSM), double emulsions were diluted 1:10 in OA and placed in Nunc Lab‐Tek eight well chambers (Thermo Fisher Scientific, USA or Ibidi µ‐Slide 18 Well #1.5 Glass Bottom Coverslips, USA) or channel slides (Ibidi sticky‐Slide VI 0.4) where dewetting took place in a confined space. GUV membranes were stained with 2.5 µм BODIPY 630/650 hydrophobic membrane selective dye unless stated otherwise. Confocal laser scanning microscopy (CLSM) micrographs were recorded on a Zeiss 880 confocal laser scanning microscope (Zeiss, Germany) using a Plan‐Apochromat 20x/0.8 M27 or water‐immersion C‐Apochromat 40x/1.2 objective. For visualizing fluorophores, a 488 nm argon laser, a 561 nm DPSS 5561‐10 laser, and a 633 nm HeNe laser were used. Images were recorded with an image size of 1024 × 1024 or 2048 × 2048 pixels with the pinhole set to one Airy unit and the bit depth set to 16 bit. Images were analyzed using Fiji image analysis software and Python scripts to measure GUV fluorescence or size.^[^ ^62^ ^]^

### Fluorescence Recovery after Photobleaching (FRAP)

For the FRAP experiments, the same confocal microscope setup was used. GUVs were fabricated with 0.1% 18:1 Liss Rhod PE (1,2‐dioleoyl‐sn‐glycero‐3‐phosphoethanolamine‐N‐(lissamine rhodamine B sulfonyl) ammonium salt) in chloroform added to the polymer organic phase. FRAP measurements of GUVs were started with ten image scans at low laser intensity (561 nm). Then a region of interest (ROI) within a 5 μm × 5 µm area was photobleached by a one frame scan at high laser intensity (561 nm). Finally, the fluorescence recovery was monitored by the acquisition of a series of 150 images with the same laser intensity as prior to bleaching. In all steps, the images were acquired using a frame size of 256 × 256 pixels and bidirectional scanning at a 33 kHz (3 × 10^−5^ s line time) line frequency scan speed, which gave a time‐lapse of 100 ms per image.

For the bleaching recovery, a uniform disk laser profile was assumed and the fluorescence recovery over time was described as:

(1) $$F\;\left(t\right)=\;\exp\left(\frac{-2\tau_{D}}{t}\right)\left[I_{0}\frac{2\tau_{D}}{t}+I_{1}\frac{-2\tau_{D}}{t}\right]$$

where *τ*_D_ = r^2^/(4D) and I_k_ are modified Bessel functions (k = 0, 1) with r being the radius of the bleached spot and D being the diffusion coefficient.

### GUV Analysis via Flow Cytometry

GUVs were stained by supplementing the OA with dibenzocyclooctyne (DBCO)‐Cy5, SYPRO Ruby Biofilm Matrix Stain, or propidium iodide (PI) via the outer aqueous phase and incubating the GUVs and dyes together for several hours (DBCO‐Cy5) to days (SYPRO Ruby, PI). The dyes and stains were diluted to a final concentration of 2 µм (DBCO‐Cy5), 10 µм (PI), and 1% (SYPRO Ruby). After incubation, GUVs were pelleted for 10 min at 1000 x g. The supernatant was discarded, and GUVs were washed twice with the same amount of fresh OA lacking dyes. The GUVs were analyzed with an Attune NxT (Thermo Fisher Scientific) flow cytometer equipped with a 488 and a 561 nm laser. For doublet exclusion, samples were gated by forward scatter (FSC)—A versus FSC‐H. FSC‐A and SSC‐A gating was used to exclude debris. Gating schemes are shown in Figure S9 (Supporting Information). Data analysis was done using FlowJo (Tree Star).

### GUV MALDI‐ToF‐MS

GUVs were washed with ultrapure Milli‐Q H_2_O by centrifugation for 10 min at 1000 x g, removal of the supernatant, and resuspension using the same volume of water. For MALDI‐ToF, 1 µL of the pellet (without resuspension) was spotted on a MALDI‐ToF target plate, together with 1 µL of the matrix. A 1:1 mix of saturated α‐cyano‐4‐hydroxycinnamic acid (CHCA) in 0.1% trifluoroacetic acid (TFA, in H_2_O) and 40 mg mL^−1^ superDHB (9:1 mixture of 2,5‐dihydroxybenzoic acid and 2‐hydroxy‐5‐methoxybenzoic acid) in 3:7 0.1% TFA: acetonitrile was used as a matrix.

For the biofilm agar‐plate samples, a sandwich approach was used; 1 µL matrix was deposited on the target plate, then, a small amount of biofilm was scraped from the agar plate using a sterile pipet tip and placed on the target plate, followed again by 1 µL of matrix. The samples were left to dry on the MALDI target plate and then analyzed using a Shimadzu MALDI‐8020 Mass Spectrometer. The resulting mzml‐files were analyzed using Python and the Pearson correlation coefficient of two data sets X and Y was calculated with the following formula:

(2) $$\rho_{X,Y}=\frac{\mathrm{cov}\left(X,Y\right)}{\sigma_{X}\sigma_{Y}}$$

where cov is the covariance, σ_X_ is the standard deviation of X and σ_Y_ is the standard deviation of Y.

### Statistical Analysis

For comparative analysis, independent two‐tailed t‐tests and one‐/two‐way Analysis of Variance (ANOVA) was used in combination with post‐hoc Tukey's honestly significant difference (HSD) test. *P* < 0.05 was considered statistically significant. The significance level of the calculated p values was indicated using asterisks: *p* > 0.05 (n.s.), *p* < 0.05 (*), *p* < 0.005 (**), and *p* < 0.0005 (***). Unless stated otherwise, no data pre‐processing was done and the mean ± standard deviation is presented. In boxplots, boxes span the interquartile range (25–75%). Statistics was done using the Python scipy.stats library.^[^ ^63^ ^]^ Statistical output of the tests is presented in Figures S23–S27 and Tables S8–S19 (Supporting Information). The 95% confidence interval (CI) is calculated using the following formula:

(3) $$\mathrm{CI}\;=\bar{x}\;\pm 1.96\frac{\sigma }{\sqrt{n}}$$

where $\bar{x}$ is the sample mean, σ the standard deviation and n the sample size.

### Data Analysis

Data analysis was done using Python. Code is available upon request.

## Conflict of Interest

The authors declare no conflict of interest.

## Author Contributions

L.H., E.C.d.S., and C.G.P. performed conceptualization. L.H., E.C.d.S., D.M., and C.‐A.S. performed the methodology. L.H. and D.M. performed the investigation. L.H. and C.‐A.S. performed visualization. D.S. and E.L. performed device fabrication. C.G.P. performed funding acquisition. L.H. and D.M. wrote the original manuscript. L.H., D.M., E.C.d.S., D.S., E.L., C.‐A.S, C.G.P. wrote review and performed editing.

## Supporting information

Supporting Information

## Data Availability

The data that support the findings of this study are openly available in zenodo at https://doi.org/10.5281/zenodo.7624093, reference number 7624093.
