# Supplementary material for: Microfluidic Giant Polymer Vesicles Equipped with Biopores for High‐Throughput Screening of Bacteria
Source: Adv Sci (Weinh). 2023 Dec 29;11(11):2307103. doi: 10.1002/advs.202307103 (PMC10953582; doi:10.1002/advs.202307103)
Supplement: Supplementary file 1 — Supporting Information [file ADVS-11-2307103-s001.pdf]

ADVANCED SCIENCE
Open Access

Supporting Information

for *Adv. Sci.*, DOI 10.1002/advs.202307103

Microfluidic Giant Polymer Vesicles Equipped with Biopores for High-Throughput Screening of Bacteria

*Lukas Heuberger, Daniel Messmer, Elena C. dos Santos, Dominik Scherrer, Emanuel Lörtscher, Cora-Ann Schoenenberger and Cornelia G. Palivan**

Microfluidic Giant Polymer Vesicles Equipped with Biopores for High-Throughput Screening of Bacteria

Lukas Heuberger[1], Daniel Messmer[1], Elena C. dos Santos[1], Dominik Scherrer[2], Emanuel Lörtscher[2,3], Cora-Ann Schoenenberger[1], Cornelia G. Palivan[1,3,4,*]

[1]*Department of Chemistry, University of Basel, Mattenstrasse 22, 4002 Basel, Switzerland*

[2]*IBM Research Europe - Zürich, Säumerstrasse 4, 8803 Rüschlikon, Switzerland*

[3]*NCCR-Molecular Systems Engineering, Mattenstrasse 24a, BPR 1095, 4058 Basel, Switzerland*

[4]*Swiss Nanoscience Institute (SNI), Klingelbergstrasse 82, 4056 Basel, Switzerland*

Supplementary Information

1 Microfluidic device

1.1 Chip Layout and Design

A Si-glass microfluidic chip was used to produce double emulsions: channel width (IA, PO, OA): 50 μm, angles between PO/OA and OA/outlet channels: 60°, angles between IA/PO channels: 30° (Figure S1). The outlet channel opens in a 60° angle up to 1 mm width and is then reduced to 300 μm before the outlet. Details on the design and etching procedure have been published previously.[1]

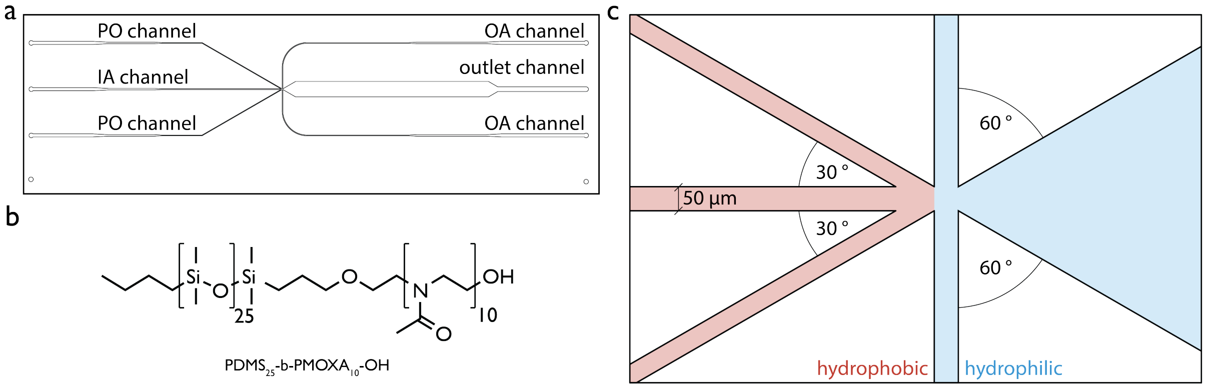

Figure S1: a) Complete design of microfluidic six-way junction on a silicon-glass microfluidic chip b) Structural formula of $PDMS_{25}$-*b*-$PMOXA_{10}$. c) Channel dimensions and angles of the six-way junction. Channel coating is used for the creation of double emulsions. For this, the inner aqueous and polymer organic channels are rendered hydrophobic using Aquapel whereas the rest is rendered hydrophilic through plasma activation and coating with polyvinyl alcohol.[1]

1.2 Throughput Calculation and Experimental Determination

A theoretical estimate of the double emulsion throughput was derived with the following equation:

$$f = \frac{v_{flow,IA}}{V_{GUV}} = 1.4 \cdot 10^3 \, Hz = 1.4 \, kHz \tag{1}$$

where $v_{flow,IA} = 3 \cdot 10^{-6} \; L \; min^{-1}$ is the typically applied inner aqueous flow velocity and $V_{GUV} = 4/3 \; r^3\pi = 3.35 \cdot 10^{-14} \; m^3$ is the GUV volume based on a double emulsion diameter of 40 μm. An IA encapsulation efficiency of $\sim$ 100 % was assumed based on previous studies.[1]

To experimentally confirm the theoretical formation rates of the double emulsion, movies of vesicle production at 2068 frames per second wer recorded using a high-speed camera. These movies were then analyzed using Python, and the double emulsions formed were detected using a Circle Hough Transform (CHT) algorithm (Figure S2a,b, Figure S6). The detected double emulsions were counted (Figure S2a), and a correction was applied to remove double emulsions detected in two consecutive frames (Figure S2b). From this, a double emulsion production rate of 319 ± 114 Hz was calculated (n=5). The discrepancy between theoretical and experimental double emulsion production rate may be explained by insufficient frame rate of the camera, low accuracy and precision of flow rate delivered by the pumps or by incomplete encapsulation of the inner aqueous phase, where not all of the inner aqueous volume is encapsulated in the double emulsion. This could be improved by lower IA flow rates, which in turn would also reduce the throughput.

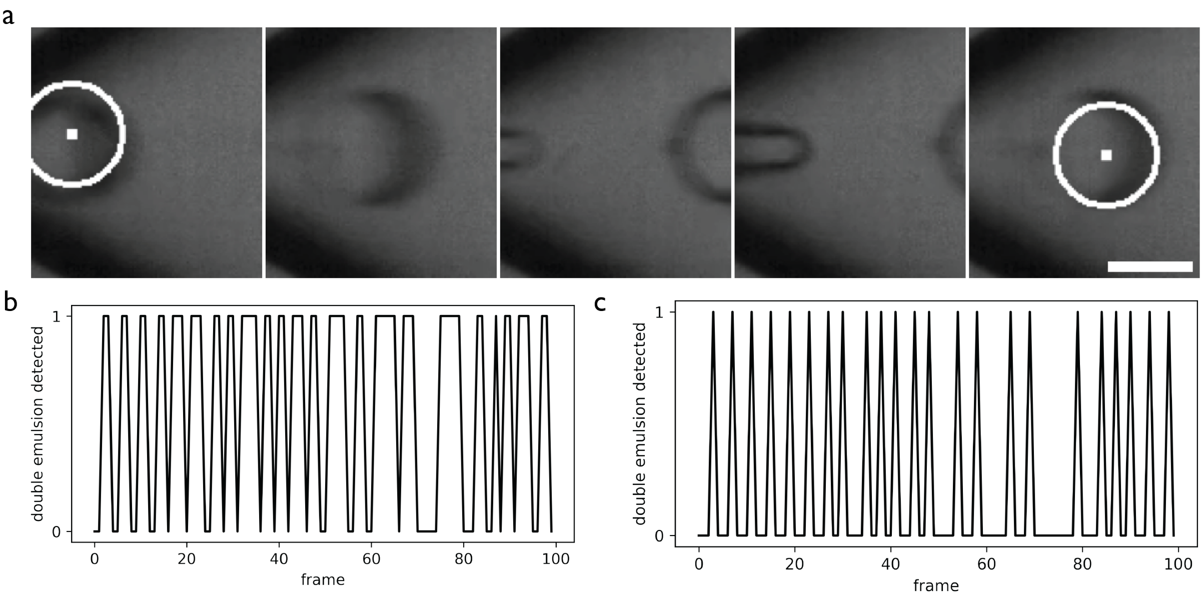

Figure S2: Images of new GUVs being produced just after the 6-way junction. Scale bar, 40 μm. 30 ms between frames. a) High frame rate images of double emulsion creation. Images were analyzed using a CHT algorithm to detect double emulsions (white circles). Scale bar, 30 μm. b) Detected double emulsions in a fraction of the total measurement (100 frames $\simeq$ 48 ms). c) Detected double emulsions after correction was applied to remove detected double emulsions in two consecutive frames.

1.3 Stability Improvement Using Pluronic F-68

The stability of diblock copolymer-based GUVs was improved with the surfactant Pluronic F-68, a triblock surfactant based on poly(propylene oxide) and poly(ethylene oxide) (PEO_{76}-PPO_{29}-PEO_{76}). To this end, different concentrations of Pluronic F-68 were compared when introduced into GUVs with a $PVA_{13,000-23,000}$-containing outer aqueous phase (data not shown) and compared to controls without Pluronic F-68 by incubation at room temperature for several days (Figure S3). It was found that a concentration of 0.1 % Pluronic F-68 had the best effect on the long-term stability of the vesicles. In addition, the outer aqueous polymer was replaced with long-chain polyethylene glycol ($PEG_{35,000}$) to improve biocompatibility. While double emulsions can be produced with outer aqueous phase only containing $PEG_{35,000}$ instead of $PVA_{13,000-23,000}$, the resulting GUVs are inherently instable and only when adding the surfactant Pluronic F-68, equal or better stability compared to $PVA_{13,000-23,000}$ outer aqueous phase GUVs can be reached.

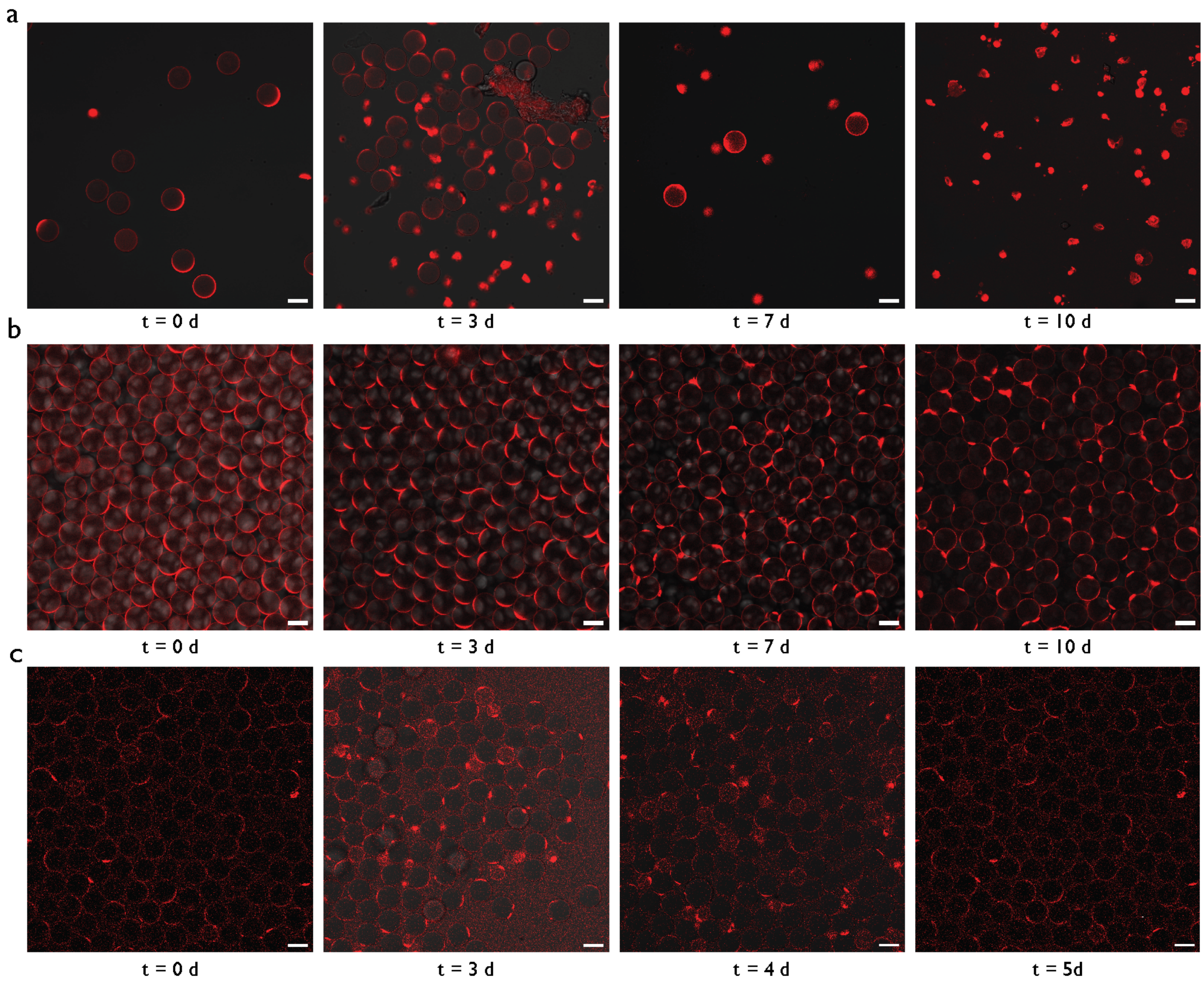

Figure S3: Influence of Pluronic F-68 on the stability of GUVs. a) GUVs created with an outer aqueous phase with 5 % $PVA_{13,000-23,000}$ and without Pluronic over 10 days at room temperature. b) GUVs created with an outer aqueous phase with 5 % $PVA_{13,000-23,000}$ and 0.1 % Pluronic F-68 over 10 days at room temperature. c) GUVs created with an outer aqueous phase with 5 % $PEG_{35,000}$ and 0.1 % Pluronic F-68 over 5 days at room temperature. Scale bars, 30 μm.

1.4 GUV Stability

To determine the stability of GUVs based on the difference between inside and outside osmolarity, a range of osmolarities were screened (Table S1) for their stability at room temperature (RT) and 37 °C.

Table S1: Measured osmolarities of IA and OAs used for testing the long-term stability of GUVs. Osmolarity values describe mean ± standard deviation of 3 measurements.

	**composition**	**NaCl [mM]**	**osmolarity [mOsmol kg^{-1}]**	$\Delta_{osmolarity}$ **(OA-IA) [mOsmol kg^{-1}]**
IA	LB, 5 % $PEG_{35,000}$	0	476.3 ± 9.5	
OA	LB, 5 % $PEG_{35,000}$, 0.1 % F-68	0	476.3 ± 5.1	0.0
OA	LB, 5 % $PEG_{35,000}$, 0.1 % F-68	50	558.0 ± 3.6	81.7
OA	LB, 5 % $PEG_{35,000}$, 0.1 % F-68	100	669.3 ± 13.1	193.0
OA	LB, 5 % $PEG_{35,000}$, 0.1 % F-68	150	803.7 ± 17.5	327.4
OA	LB, 5 % $PEG_{35,000}$, 0.1 % F-68	200	851.3 ± 16.4	375.0

The resulting GUVs were imaged over several days and their stability was determined relative to their initial vesicle concentration. Imaging was done in urinalysis slides (Fast Read 102 counting chambers) on a Olympus EP50 microscope. 20 μM calcein was added to the IA of the vesicles during production for automated image analysis. Figure S4 shows the highest stability was found in samples containing 0, 50 or 150 mM NaCl. Additionally, no significant difference in stability was observed when exposing GUVs to 37 °C. Further, in most combinations, an initial drop in GUV number of 20-40 % after 1 day of incubation is observed with a plateau afterwards, possibly due to an initial instability of the GUVs or the dispersal method used before imaging.

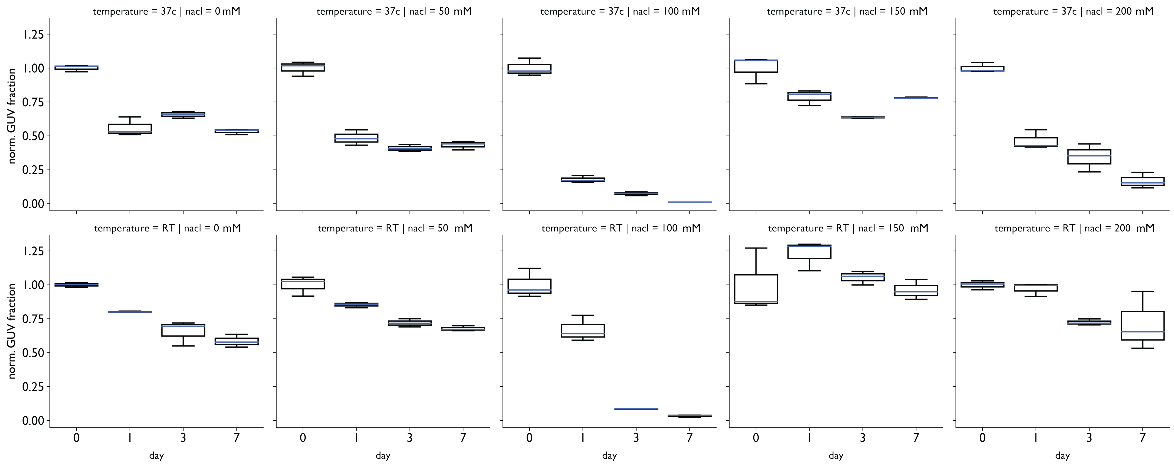

Figure S4: Stability of GUVs made with different NaCl concentration outer phases (OA) evaluated for up to 7 days at 37 °C (top row) and room temperature (RT, bottom row). Stability is calculated by number of GUVs relative to the initial number after production (day 0) (n=3).

1.5 Hydrophobic Pocket after Dewetting

During the evaporation of the organic phase, the volatile organic solvents (a 3:2 mix of hexane and chloroform) will be drawn towards the top of the forming GUV, leaving behind a polymer pocket that can be stained with the lipophilic dye BODIPY 630/650 (Figure S5).

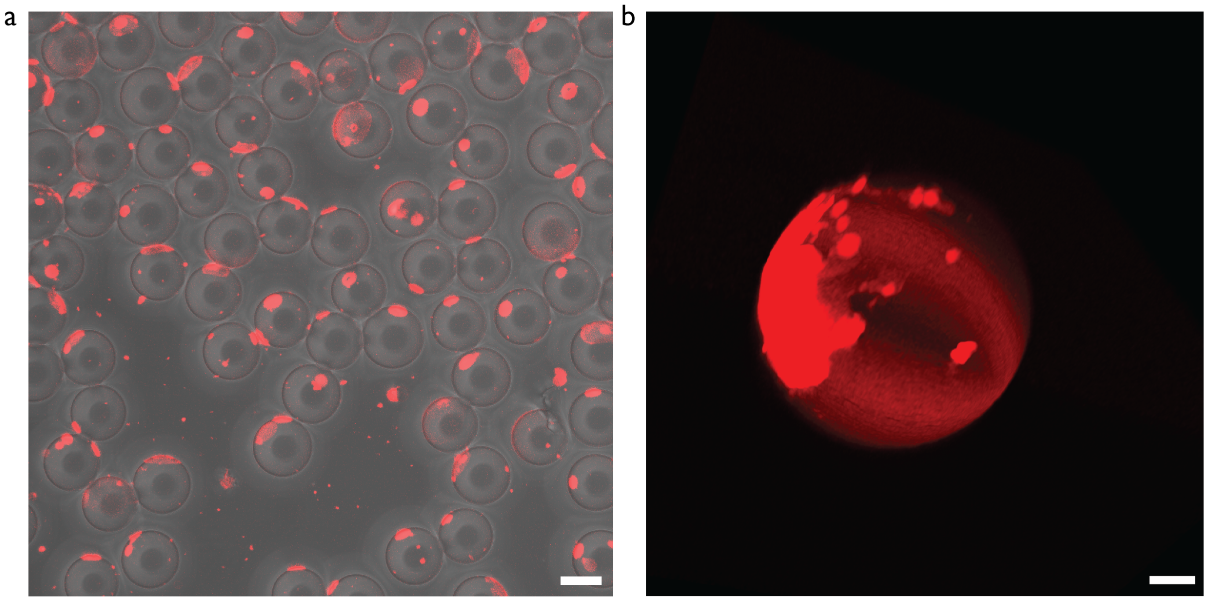

Figure S5: a) Fluorescence micrograph of a maximal z-projection of a z-stack of GUVs stained with BODIPY 630/650 to visualize polymer pockets after dewetting. Scale bar, 30 μm. b) 3d reconstruction of a z-stack of a GUV stained with BODIPY 630/650. Scale bar, 7 μm.

1.6 Measuring GUV Diameters

To determine the diameter and distribution of produced GUVs, microscopic images of a set of GUVs were recorded using the CLSM. The micrographs were analyzed using a Python script to measure the diameters of the GUVs. The script is based on the CHT algorithm from the previous section and can detect circles in images with high precision. Using this algorithm, circles were detected either in the transmission channel or frequently in the BODIPY 630/650 channel (Figure S6). The detected circles were measured and a size histogram was generated. The diameters agree with a normal distribution with a relative standard deviation of 1.6 %.

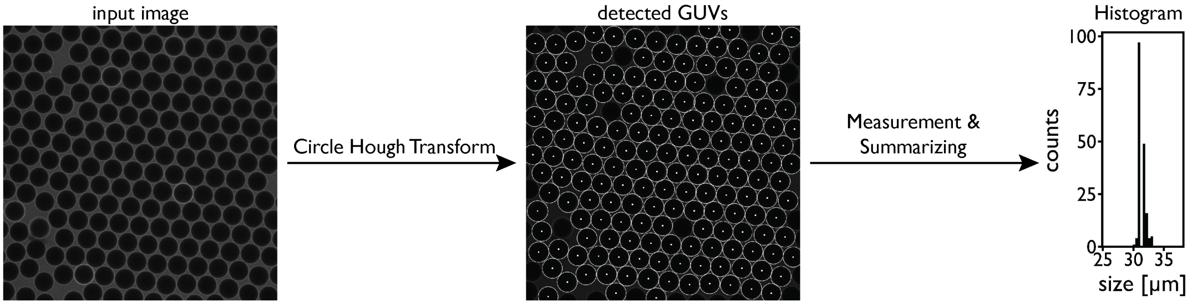

Figure S6: Schematic of workflow used to measure GUV diameters using Circle Hough Transform.

2 Characterizing Properties and Functionalities of GUVs

2.1 Influence of Microfluidic Shear Stress on Bacterial Survival

To determine the influence of the microfluidic processing on bacterial viability, the effects of flowing bacteria through microfluidic channels were investigated. For this purpose, the viability of GFP-expressing *E. coli* (control; Figure S7a) was compared to *E. coli* coincubated with the polymer PDMS-*b*-PMOXA (Figure S7b), *E. coli* subjected to low shear stress by flowing through the microfluidic chip (Figure S7c), and *E. coli* encapsulated in GUVs (Figure S7d). To assess the bacterial viability, propidium iodide (PI), a fluorescent dye that stains only dead bacteria, was used. From the micrographs in Figure S7, it is evident that in control (a) very few bacteria are dead (white arrows) while the majority show only green fluorescence. When *E. coli* and polymer are mixed, few bacteria are visible, but most show green fluorescence only (Figure S7a, green arrows) while a few fluoresce also in the PI channel (white arrows), indicating their death. Similarly, no dead bacteria were detected in bacteria loaded-GUVs that passed through microfluidic channels, indicating that shear forces during microfluidic flow do not affect bacterial viability inside GUVs. Finally, when bacteria are encapsulated in GUVs, they are predominantly viable (green) and only few (white arrows) are dead (red and green). This indicates that encapsulating bacteria in GUVs in microfluidic flow has no adverse effect on bacteria viability. To ensure that the bacteria were able to grow after being exposed to low shear, the growth of GFP-*E. coli* was compared (Figure S7e). *E. coli* in LB + 5 % $PEG_{35,000}$ (corresponding to LB inner aqueous phase, IA) and LB, 5 % $PEG_{35,000}$, 100 mM NaCl, 0.1 % Pluronic F-68 (corresponding to outer aqueous phase, OA) were compared to GFP-*E. coli* flowed through the microfluidic chip with all three liquid phases (IA, PO, and OA) in the absence of double emulsion formation but including the polymer dissolved in 3:2 hexane:chloroform. Samples were incubated overnight at 37 °C and analyzed the next day by flow cytometry. Figure S7e shows no significant difference in GFP or PI fluorescence among the three samples. Slightly wider peaks were observed in the microfluidic-treated samples (red) but this is mainly due to dilution of the bacteria during microfluidics. Im-

portantly, the *E. coli* exposed to shear do not show increased PI fluorescence compared to the IA and OA control, confirming our results in Figure S7c and Figure S7d and demonstrating no adverse effect of the 3:2 hexane:chloroform on bacterial growth due to a high dilution of the PO phase.

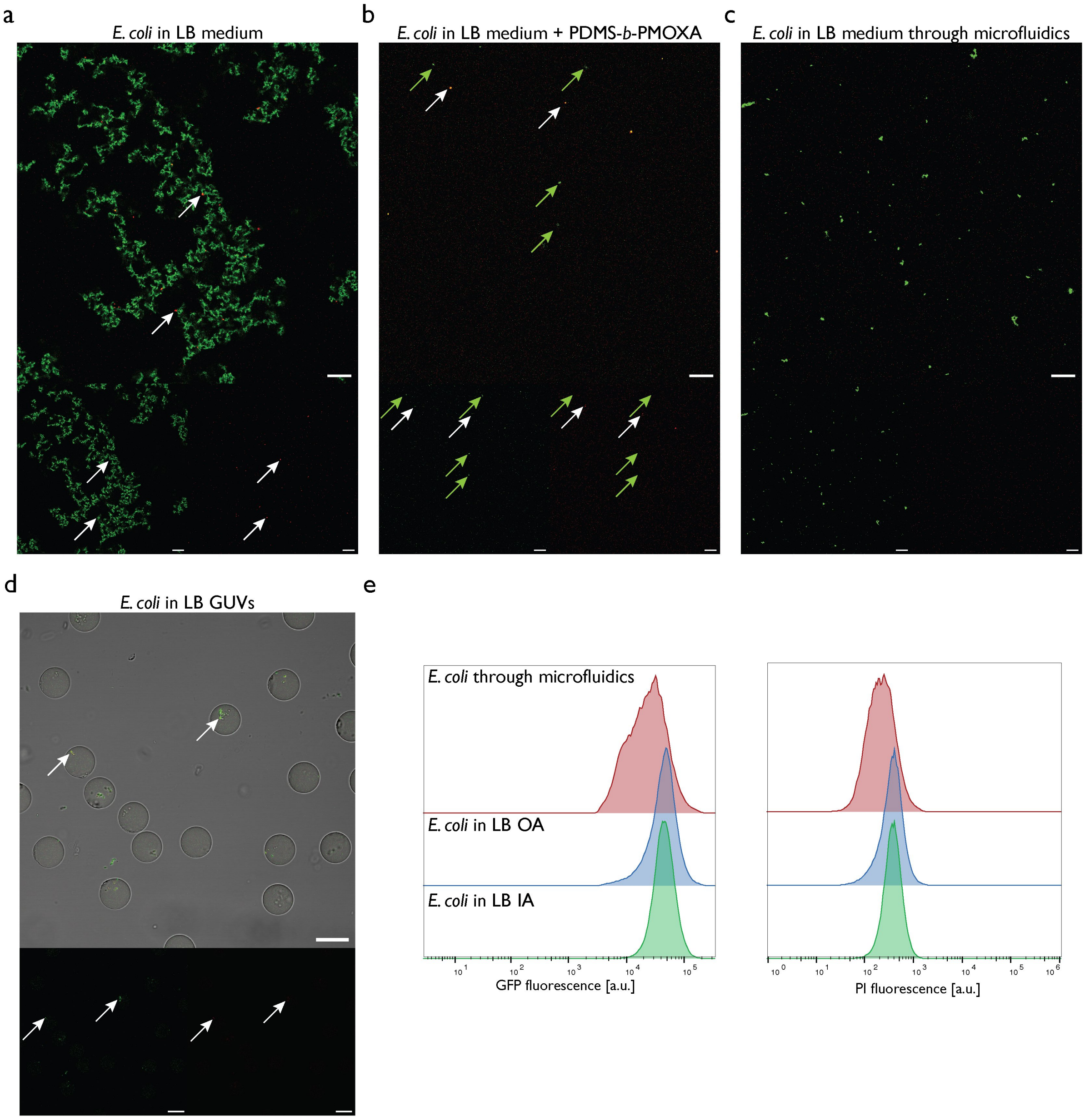

Figure S7: Fluorescence micrographs of GFP-expressing *E. coli* (green) stained with propidium iodide (red). Merged image shown on top and single channel images shown at the bottom (left: GFP, right: PI). a) Untreated *E. coli*, b) *E. coli* conincubated with PDMS-*b*-PMOXA polymer, c) *E. coli* passed through a microfluidic chip, and d) *E. coli* encapsulated in PDMS-*b*-PMOXA-GUVs. Scale bars, 30 μm. e) Flow cytometric analysis of GFP-expressing *E. coli* in IA and OA media and after microfluidic treatment. Bacteria were incubated overnight at 37 °C and analyzed the next day.

2.2 Theoretical Calculation of Bacteria/GUV

To validate the number of encapsulated bacteria per GUV, a theoretical maximum loading capacity of bacteria per GUV was calculated (Figure S8). A loading efficiency of 100 % was assumed and the encapsulated bacteria per GUV were calculated depending on the bacterial input concentration for the OD_{600} values used in Figure 3 and on the GUV size.

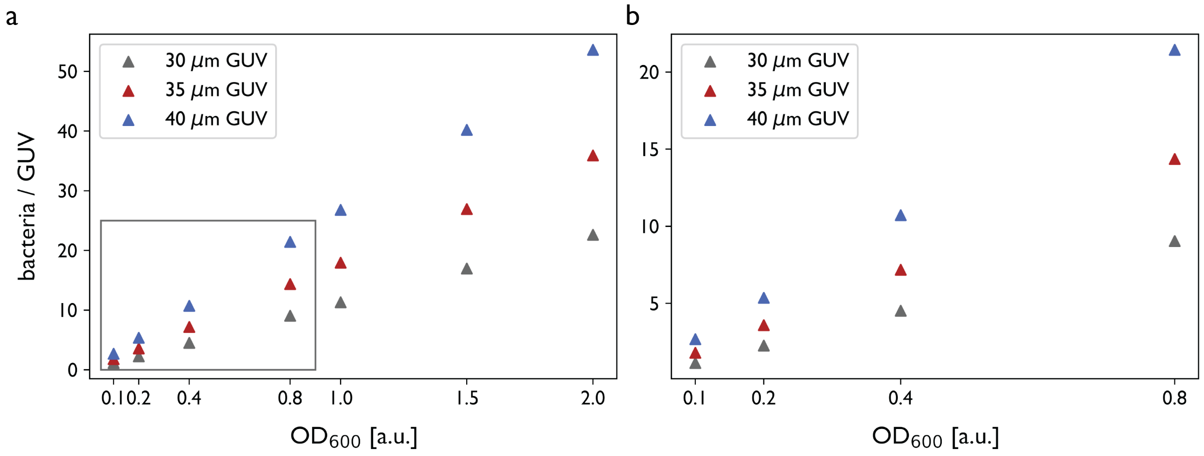

Figure S8: Calculated number of bacteria per GUV depending on the bacterial concentration in the inner aqueous phase. a) Full range of bacteria concentration tested in Figure 2, b) zoom-in on cutout in a).

2.3 Flow Cytometry Gating Strategies

For doublet exclusion, the samples were gated by forward scatter (FSC) area versus height (FSC-A vs. FSC-H, Figure S9a), optionally followed by side scatter (SSC)-A versus SSC-H (Figure S9b). When only singlet cells remained, gating was done by fluorescence using unstained controls.

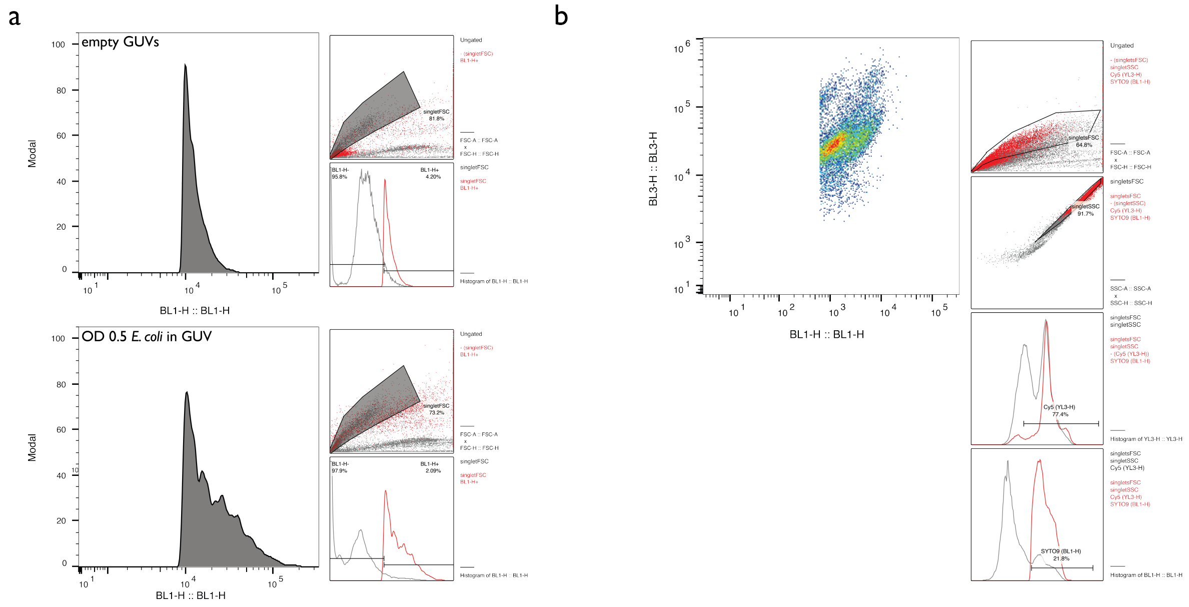

Figure S9: Representative gating strategies of Figures a) 3f and b) 6g.

2.4 Growth Curves of *E. coli* and *B. subtilis* in (modified) LB and MSgg media

As the growth of bacteria depends on the culture medium, the growth of *E. coli* and *B. subtilis* in the different media used for GUV cultures were evaluated. Specifically, LB and MSgg minimal media were compared and the effect of different additives on the media was evaluated. $PEG_{35,000}$ and $PVA_{13,000-23,000}$ were used to increase the viscosity of the media, and 100 mM sucrose was tested because it is commonly used to increase density in vesicles. Bacteria were grown in a microtiter plate for 16 hours at 37 °C with continuous shaking. The OD_{600} was measured every 15 minutes. Figure S10 shows the growth behavior of *E. coli* (Figure S10a) and *B. subtilis* (Figure S10b) for LB and MSgg media. A clear growth difference is observed between the nutrient-rich medium LB and the nutrient-poor medium (MSgg), where the OD_{600} maximum is reached only in the presence of sucrose. In LB-media, bacterial growth differs in media supplemented with $PEG_{35,000}$ and $PVA_{13,000-23,000}$. In $PEG_{35,000}$-supplemented media, growth is still retarded compared to the control, but PVA has a stronger retarding effect, especially in *B. subtilis* cultures. This effect is even more pronounced in MSgg medium, where PVA almost completely

inhibits growth. Based on these results, we concluded that $PEG_{35,000}$ should be used as a viscosity-increasing agent in bacterial encapsulation to ensure proper growth.

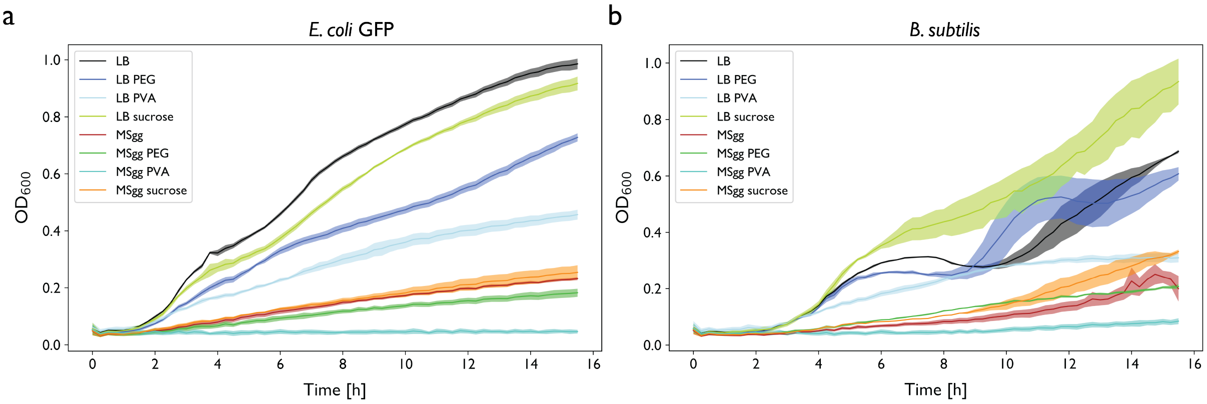

Figure S10: Growth curves of a) GFP-expressing *E. coli* and b) *B. subtilis* over 16 h at 37 °C in different nutrient rich (LB) and minimal (MSgg) media.

2.5 Permeability of GUVs to Small Molecules

To determine the permeability of the produced PDMS-*b*-PMOXA-GUVs for small molecules without additional permeabilization, their permeability for ions and small dye molecules was investigated. Using the Ca^{2+}-sensitive dye CalciumGreen-5N (CaGreen), membrane permeability was determined for a representative ion (Ca^{2+}). CaGreen was encapsulated at a concentration of 7.5 μM by addition to IA during vesicle preparation. Only after addition of melittin does the fluorescence intensity increase, indicating that the GUVs present are impermeable to small ions but can be permeabilized with melittin (Figure S11a). Similarly, empty GUVs were prepared and the low molecular weight dye calcein (622.55 Da) was added to the outside and incubated with the GUVs for one hour. No significant increase in fluorescence was measured in the lumen of GUV for calcein either (Figure S11b).

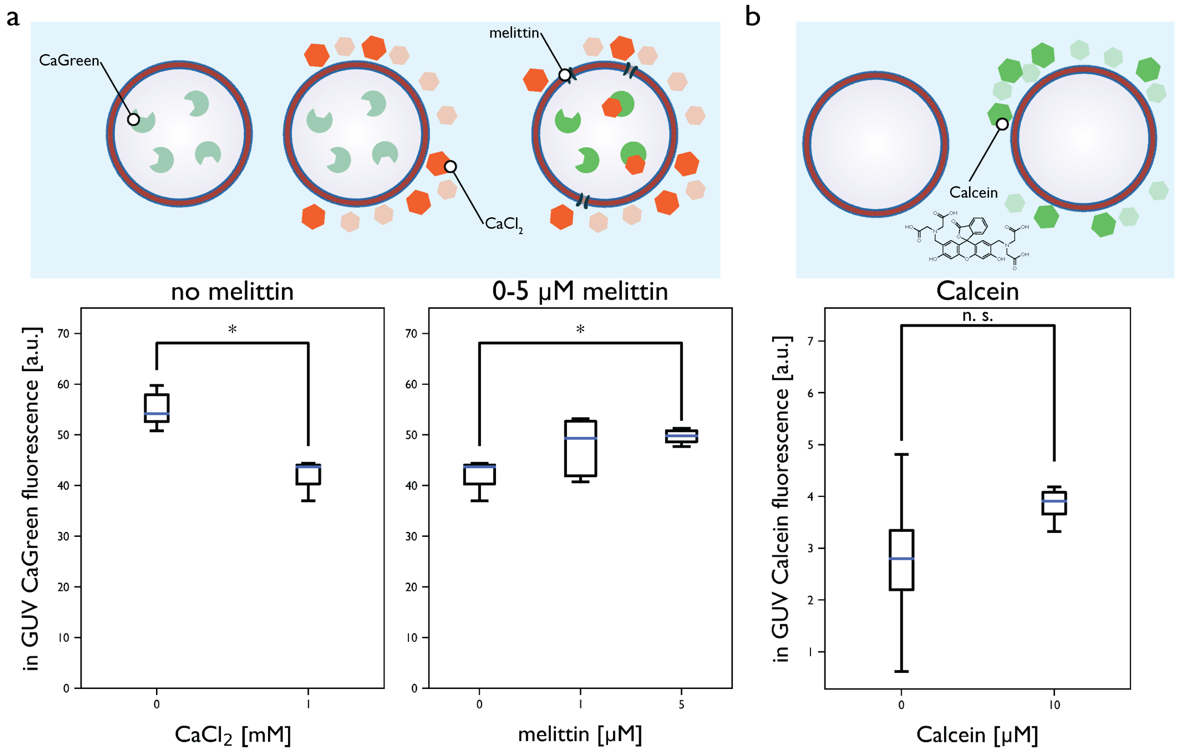

Figure S11: a) Investigation of GUV membrane permeability to Ca^{2+}-ions measured indirectly via the fluorescence of the Ca^{2+}-sensitive dye CaGreen. CaGreen fluorescence is measured with and without 1 mM $CaCl_2$ present outside of the GUVs (left panel) and with 1 mM $CaCl_2$ and different concentrations of melittin present (right panel). n=15-70 GUVs/condition. b) Investigation of GUV permeability to small molecules such as the small molecule dye calcein. Calcein fluorescence is measured inside of the GUV before and after addition of 10 μM calcein to the outside. n=40-113 GUVs/condition.

3 Permeability-Engineering of GUVs by Melittin Pores

Melittin (a pore forming peptide) was added to the outer aqueous phase (OA) during the formation of the double emulsion. To evaluate whether membrane permeabilization of the PDMS-*b*-PMOXA bilayer was reached, the enzyme β-galactosidase (β-Gal) was encapsulated in GUVs via addition to the inner aqueous phase (IA). Fluorescein di(beta-D-galactopyranoside (FDG, 656.6 Da) was added as a substrate to show the influx of molecules into the GUVs (Figure S12a). The presence of β-galactosidase is associated with an increase in fluorescence within the GUV due to the conversion of the nonfluorescent substrate FDG to fluorescein (Figure S12b). Figure S12c shows a clear difference in fluorescence within GUVs upon addition of FDG in melittin-permeabilized GUVs com-

pared with nonpermeabilized GUVs. GUVs permeabilized via melittin addition to the outer aqueous phase (OA, Figure S12d below) showed a significant increase in fluorescence inside and outside the GUVs, indicating that the converted fluorescent dye also exits the GUVs through melittin pores. No increase in fluorescence was observed upon permeabilization of GUVs via the internal aqueous phase (IA, Figure S12d above), indicating unsuccessful pore formation upon addition of melittin from the inside. The ineffective pore formation could be due to membrane curvature of GUV and was also observed for membrane pore OmpF.[1]

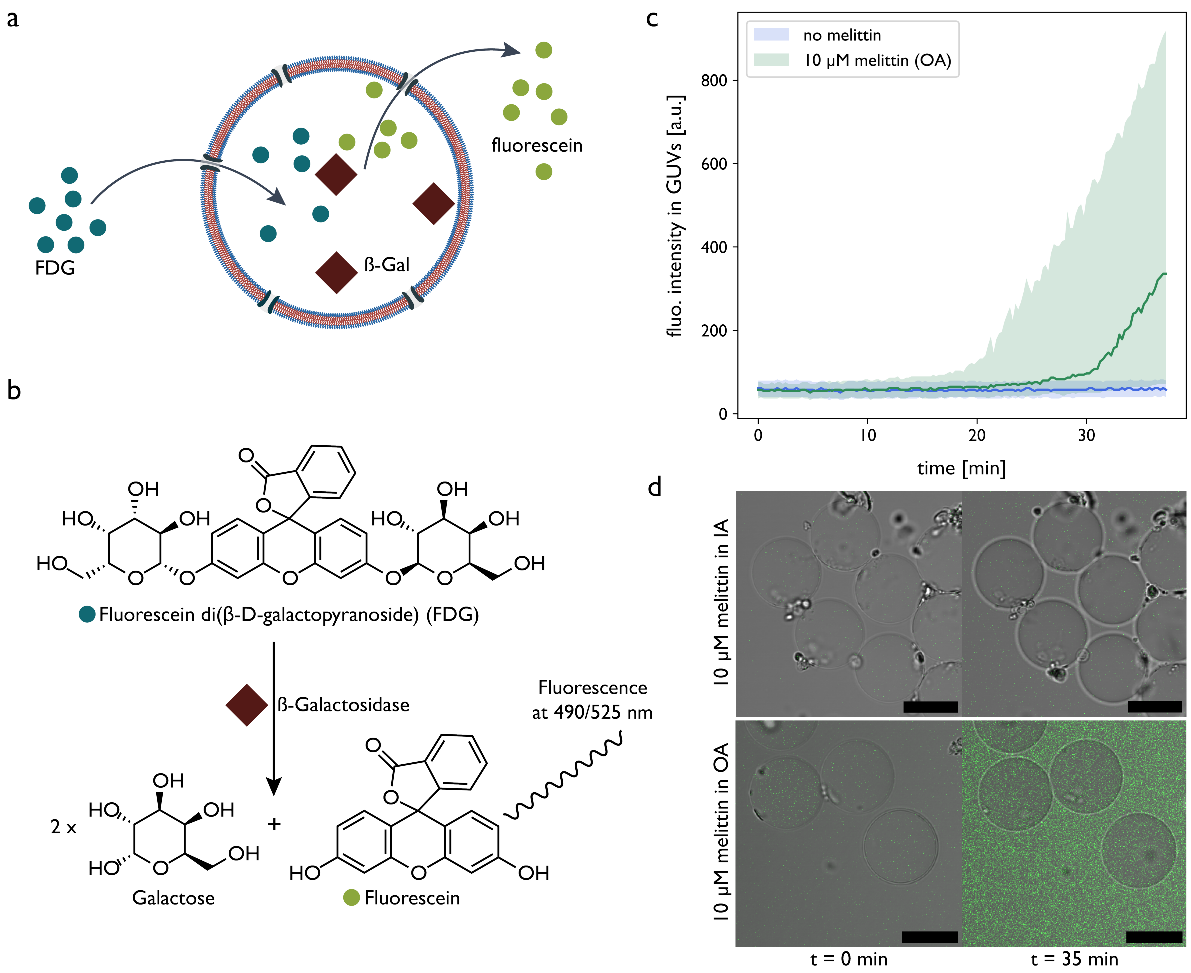

Figure S12: a) Schematic representation of the conversion of fluorescein di(beta-D-galactopyranoside) (FDG) to fluorescein by β-galactosidase (β-Gal). b) Reaction diagram of β-Gal-mediated hydrolysis of FDG to the green fluorescent product fluorescein. c) Fluorescence measured in GUV-lumen after addition of FDG to melittin-permeabilized (green) and non-permeabilized GUVs ($n = 18$ GUVs per condition). Line represents mean and shaded region represents 25 resp. 75 % confidence interval. d) Fluorescence micrographs of GUVs permeabilized from inside (top) and outside (bottom) after FDG addition (left) and after 35 min reaction time (right). Scale bars, 30 μm.

3.1 GUV Stability upon Permeabilization

The stability of GUVs was also determined upon addition of melittin in different concentrations (1, 5, 10 μM in OA) and at different incubation temperatures (room temperature (RT) and 37 °C). The influence of melittin on the osmolarity was measured and is depicted in Table S2 and Figure S13.

Table S2: Measured osmolarities of IA and OAs used for testing the long-term stability of GUVs. Osmolarity values describe mean ± standard deviation of 3 measurements.

	**composition**	**NaCl [mM]**	**melitin [μM]**	**osmolarity [mOsmol kg^{-1}]**	$\Delta_{osmolarity}$ **(OA-IA) [mOsmol kg^{-1}]**
IA	LB, 5 % $PEG_{35,000}$	0	0	476.3 ± 9.5	
OA	LB, 5 % $PEG_{35,000}$, 0.1 % F-68	0	0	476.3 ± 5.1	0.0
OA	LB, 5 % $PEG_{35,000}$, 0.1 % F-68	0	1	466.3 ± 10.4	-10.0
OA	LB, 5 % $PEG_{35,000}$, 0.1 % F-68	0	5	474.3 ± 12.7	-2.0
OA	LB, 5 % $PEG_{35,000}$, 0.1 % F-68	0	10	488.0 ± 14.2	12.0
OA	LB, 5 % $PEG_{35,000}$, 0.1 % F-68	100	0	669.3 ± 13.1	193.0
OA	LB, 5 % $PEG_{35,000}$, 0.1 % F-68	100	1	664.0 ± 11.8	167.7
OA	LB, 5 % $PEG_{35,000}$, 0.1 % F-68	100	5	658.7 ± 13.3	182.4
OA	LB, 5 % $PEG_{35,000}$, 0.1 % F-68	100	10	695.7 ± 15.0	219.4

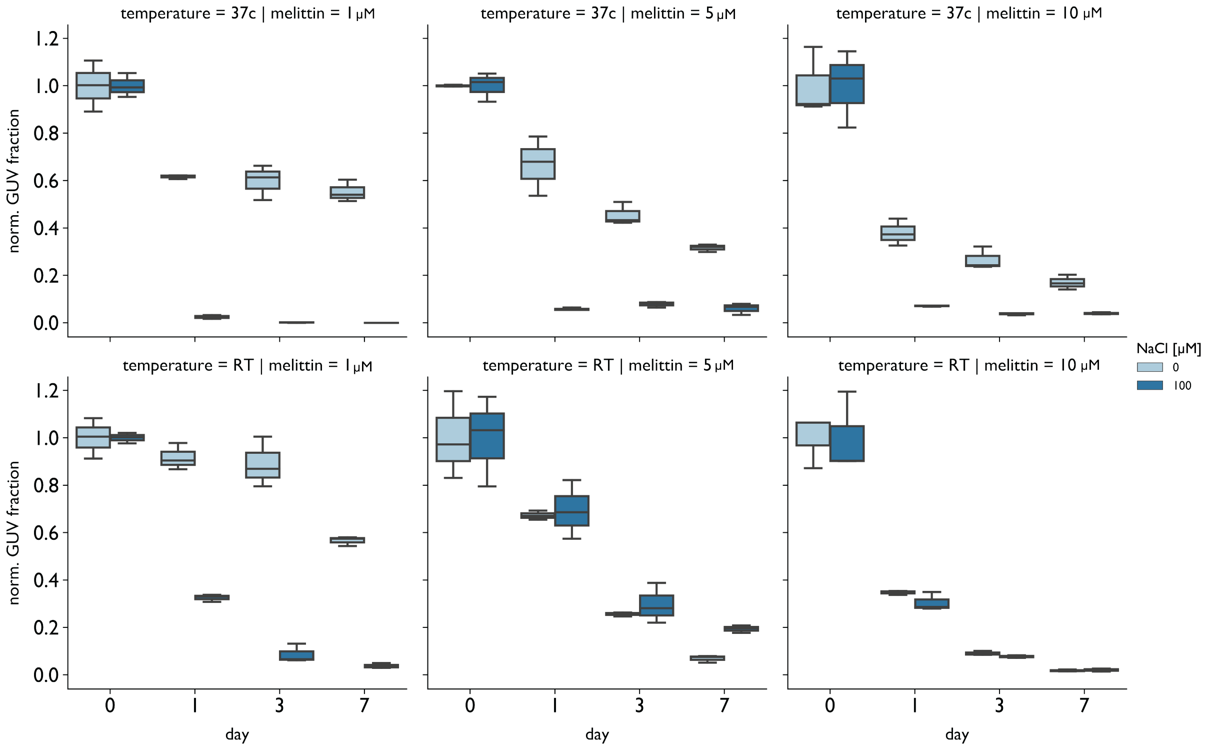

Figure S13: Stability of GUVs made with different melittin concentration outer aqueous phases evaluated for up to 7 days at 37 °C (top row) and room temperature (RT, bottom row). The stability of GUVs made with OA containing no additional salt (light blue) or 100 mM NaCl (dark blue) was compared. Stability is calculated by number of GUVs relative to the initial number after production (day 0) (n=3).

3.2 GUV Permeability to Bacterial Stains

To validate the diffusion of dyes used for bacterial staining through a GUV membrane for *post-hoc* staining of bacteria encapsulated in GUVs, the fluorescence of bacteria stained with SYTO 9 and PI dyes was compared between internal and external dye addition. *B. subtilis* was added to the GUV IA at an OD_{600} of 0.8 and SYTO 9 was added at a concentration of 5 μM either to the IA or to the tube after collection. Melittin-permeabilized GUVs were incubated overnight at 37 °C, and fluorescence of individual encapsulated bacteria was measured the next day using fluorescence micrographs. No significant difference in the fluorescence intensity of the encapsulated bacteria was detected as a function of the administration method (Figure S14a). To determine the permeability of melittin-permeabilized GUVs to PI, dead bacteria were encapsulated in GUVs. For this purpose, *E. coli* were grown and then heated to 95 °C for 15 min and then diluted to an OD_{600}

0.7. PI was added to either the IA phase or the OA after collection for 1 h or for 24 h at a concentration of 10 μM. GUVs were also incubated overnight at 37 °C, and fluorescence intensity was measured after 24 h using fluorescence micrographs. Similarly, no significant difference in PI intensity of dead bacteria was observed regardless of the addition method (Figure S14b). To validate the applicability of staining *B. subtilis* for growth assays, the influence of staining bacteria with SYTO 9 was investigated in a growth assay. By comparing the growth profiles of stained and unstained *B. subtilis* in LB (Figure S14c) and LB supplemented with 5 % $PEG_{35,000}$ (Figure S14d), the influence of SYTO 9 staining on bacterial growth can be investigated. While the addition of SYTO 9 causes a slight lag in the growth, both conditions reached similar plateaus and no growth inhibition was observed, validating the applicability of SYTO 9 staining for *B. subtilis* growth monitoring in GUVs.

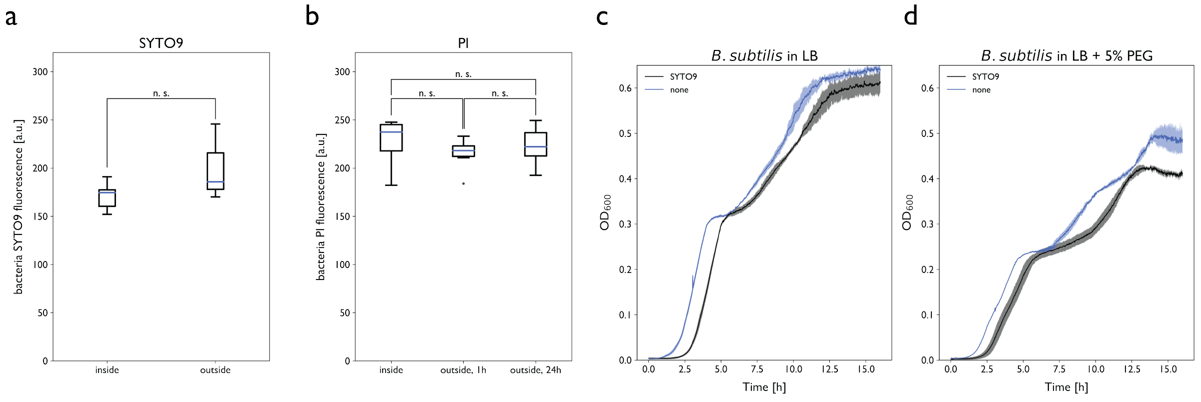

Figure S14: a) Nucleic acid staining SYTO 9 fluorescence intensity of *B. subtilis* in GUVs after overnight incubation with SYTO 9 added to the IA phase (inside) or after GUV production (outside) (n=3-7). b) Dead staining PI fluorescence intensity of dead *E. coli* in GUVs after overnight incubation with PI added to the IA phase (inside) or after GUV production for 1 h (outside, 1 h) or 24 h (outside, 24 h) (n=7). c) Growth curves of *B. subtilis* with and without SYTO 9 stain in LB medium (n=3). d) Growth curves of *B. subtilis* with and without SYTO 9 stain in LB medium supplemented with 5 % $PEG_{35,000}$ (n=3).

3.3 Minimal Inhibitory Concentration Determination of *E. coli* and *B. subtilis*

3.3.1 Kanamycin MIC for *E. coli* and *B. subtilis* in LB and MSgg medium

To determine the resistance of the strains of GFP-expressing *E. coli* and *B. subtilis* to the antibiotic kanamycin in the media used in this study, broth microdilution assays were performed and the growth of the bacteria in media supplemented with various amounts of kanamycin was monitored by monitoring OD_{600}. From the growth curves, the fractional area (FA) under the OD_{600}/time curves was calculated relative to the control without antibiotics (Figure S15). Using a Python script, the data was background corrected and the areas under the curves were calculated using the trapezoidal rule. The obtained FA was fitted to a modified Gompertz function (four-parameter dose-response model):

$$FA_{OD_{600}} = A + C * e^{-e^{B(x-M)}} \tag{2}$$

where A = minimum FA(OD_{600}) (≈ 0), B = slope factor, C = distance between upper and lower asymptote (≈ 1), M = log antibiotics concentration at inflection point, and x = log of the antibiotics concentration.[2–4] The minimal inhibitory concentration (MIC) is defined as:

$$MIC = 10^{M+\frac{1}{B}} \tag{3}$$

and resulted in MIC values listed in Table S3. Parameter values used for curve fitting of the dose response curves shown in Figure S15 can be found in Table S3.

Table S3: Parameters used for curve fitting of antibiotic dose-response curves and calculated MIC values.

Condition	A	B	C	M	calculated MIC [μg mL^{-}1]
B. subtilis in LB	0.94	0.81	1.60	-0.19	0.08
B. subtilis in MSgg	0.94	1.48	0.07	-0.26	1.30
E. coli in LB	0.97	0.90	0.49	-0.17	1.53

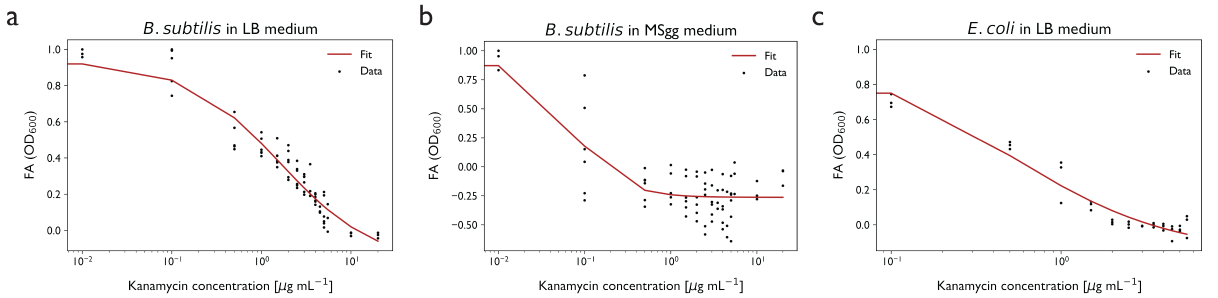

Figure S15: Dose response curve of a+b) *B. subtilis* and c) GFP-*E. coli* in LB and MSgg medium. Observed growth (black dots) and fitted four parametric logistic dose-response model (red).

3.3.2 Ampicillin and Vancomycin MIC of GFP-*E. coli* in LB

MIC values were further calculated for the antibiotics ampicillin and vancomycin in LB medium supplemented with 5 % $PEG_{35,000}$. As shown for kanamycin, the fractional areas below the curves were calculated from the growth curves and plotted against the antibiotic concentration (Figure S16). Using the Gompertz function (Equation (2)), growth curves were fitted and the MIC was calculated using equation (Equation (3)). Parameter values used for curve fitting of the dose response curves shown in Figure S16 and calculated MIC values are presented in Table S4.

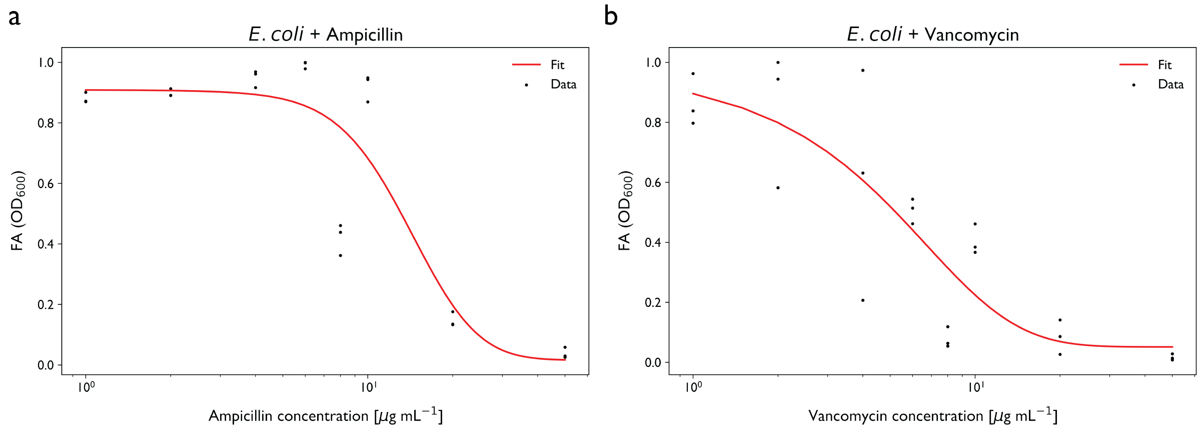

Figure S16: Dose response curve of GFP-*E. coli* in LB medium exposed to a) ampicillin and b) vancomycin. Observed growth (black dots) and fitted four parametric logistic dose-response model (red).

Table S4: Parameters used for curve fitting of antibiotic dose-response curves and calculated MIC values.

Condition	A	B	C	M	calculated MIC [μg mL^{-}1]
E. coli + Ampicillin	0.91	-0.18	-0.89	11.76	17.3
E. coli + Vancomycin	1.20	-0.23	-1.15	2.22	6.5

3.4 3D Dye Colocalization in Biofilm-GUVs

Functionalization of the GUV membrane is an excellent tool for immobilizing dyes or other functional groups on the membrane. For this purpose, the azide-terminated polymer $PDMS_{27}$-*b*-$PMOXA_8$-PEG_3-N_3, which co-assembles into a membrane with $PDMS_{25}$-*b*-$PMOXA_{10}$, was used.[5] Using strain-promoted alkyne-azide cycloaddition between an azide and dibenzocyclooctyne (DBCO, Figure S17a), the blue fluorescent dye Cy5 was covalently immobilized to the GUV outer membrane. By combining dye functionalization of the membrane with other fluorescent dyes to stain bacteria (SYTO 9, green) and biofilm matrix components (SYPRO Ruby Biofilm Matrix Stain, red), colocalization of bacteria, biofilm matrix, and the polymer membrane was done (Figure S17b,c). Colocalization analysis was carried out by collecting z-stacks of GUVs over the course of 3 days and evaluating Mander's correlation coefficient M1 for different dyes per z-stack (Figure S17d). This allowed us to determine the average dye colocalization per GUV over time (Figure S17e). The correlation of SYTO 9 and SYPRO Ruby allows colocalization of bacteria and biofilm matrix. Over time, this correlation significantly increases, indicating colocalization of biofilm matrix components with bacteria. Similarly, colocalization of bacterial staining and membrane staining also signficantly increases over time. This can be interpreted as movement of bacteria to the membrane where they deposit biofilm matrix components.

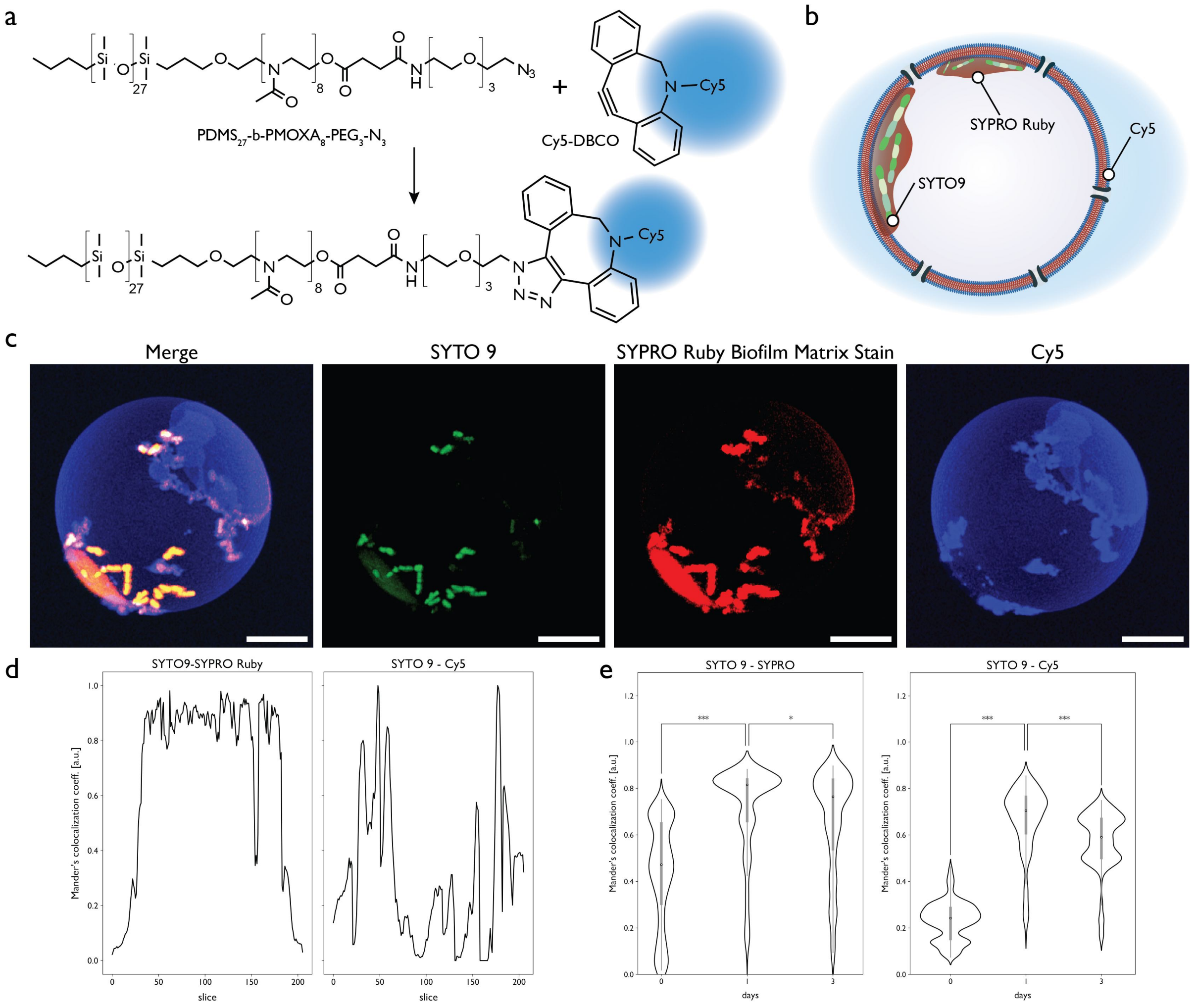

Figure S17: a) Reaction scheme of strain-promoted alkyne-azide cycloaddition between an azide-terminated PDMS-*b*-PMOXA polymer and Cy5-dibenzocyclooctyne (Cy5-DBCO). b) Schematic representation of dye localization in *B. subtilis*-encapsulating GUVs. c) Maximal z-projection of z-stack of a *B. subtilis*-encapsulating GUV after 3 d incubation. Scale bars, 10 μm. d) Exemplary Mander's colocalization coefficient in a z-stack fluorescent micrograph of a *B. subtilis*-encapsulating GUV after 3 d incubation. e) Violin plots of Mander's M1 colocalization coefficient over time (n $\geq$ 9 per condition).

The post-hoc Tukey HSD results for the comparison of the correlation coefficients for SYTO 9 and SYPRO Ruby (Table S5) and SYTO 9 and Cy5 (Table S6) are presented below.

Table S5: Tukey HSD results of the comparison of Mander's M1 colocalization coefficients over time of SYTO 9 and SYPRO Ruby Biofilm Matrix Stain (Figure S11e).

**group1**	**group2**	**meandiff**	**p-adj**	**lower**	**upper**	**reject**
d0	d1	0.2717	0	0.2227	0.3208	TRUE
d0	d3	0.2172	0	0.1682	0.2663	TRUE
d1	d3	-0.0545	0.0251	-0.1036	-0.0054	TRUE

Table S6: Tukey HSD results of the comparison of Mander's M1 colocalization coefficients over time of SYTO 9 and Cy5 (Figure S11e).

**group1**	**group2**	**meandiff**	**p-adj**	**lower**	**upper**	**reject**
d0	d1	0.4305	0	0.4022	0.4588	TRUE
d0	d3	0.3411	0	0.3128	0.3694	TRUE
d1	d3	-0.0894	0	-0.1176	-0.0611	TRUE

3.5 Membrane Fluidity of Hybrid Membranes

The incorporation of lipids in polymeric membranes can alter their fluidity, as lipid membranes tend to have higher fluidities than polymer membranes.[6] By adding 1-palmitoyl-2-oleoyl-sn-glycero-3-phosphocholine (POPC) to the PO during GUV formation, hybrid GUVs can be created and the incorporation of lipids, e.g., from depositions of biofilm matrix in polymeric membranes, can be mimicked. Using fluorescence recovery after photobleaching (FRAP), membrane fluidity of pure polymer GUVs and GUVs supplemented with 1 or 5 % POPC was compared (Figure S18). A significant increase of membrane fluidity was already visible at 1 % POPC in the membrane, indicating the successful incorporation of lipids into the polymer membrane.

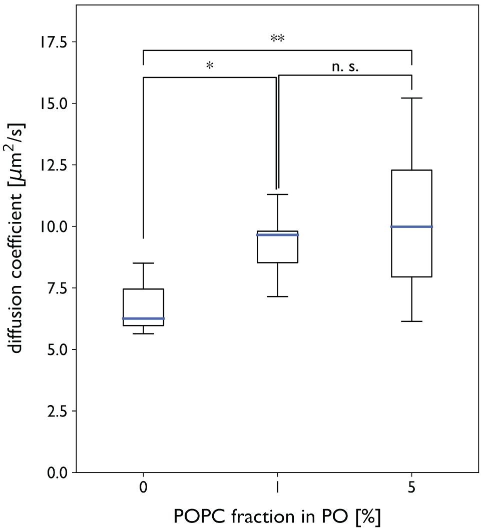

Figure S18: Diffusion coefficient of PDMS-*b*-PMOXA GUVs supplemented with 1-5 % of the lipid 1-palmitoyl-2-oleoyl-sn-glycero-3-phosphocholine (POPC) (n=10).

The post-hoc Tukey HSD results for the comparison the membrane fluidity with different POPC concentrations are presented below (Table S7).

Table S7: Tukey HSD results of the comparison of Mander's M1 colocalization coefficients over time of SYTO 9 and Cy5 (Figure S18).

group1	group2	meandiff	p-adj	lower	upper	reject
1% POPC	5% POPC	0.9349	0.5597	-1.2977	3.1676	FALSE
1% POPC	control	-2.5822	0.021	-4.8148	-0.3495	TRUE
5% POPC	control	-3.5171	0.0016	-5.7498	-1.2844	TRUE

3.6 Analysis of GUV Contents by MALDI-ToF

Matrix-assisted laser desorption/ionization (MALDI) coupled with time-of-flight mass spectrometry (MALDI-ToF MS) can be used for the detection of biomolecules such as proteins and peptides and is commonly used for mass spectrometry of bacteria.[7–9] However, the choice of buffer has a great impact on the signal quality and can also completely suppress the analyte signal.[10] To improve the MALDI-ToF signal a washing procedure was established. Melittin was encapsulated in GUVs at a concentration of 25 μM as a test target. For comparison, melittin solutions in Milli-Q H_2O, inner aqueous phase (MSgg

+ 5 % $PEG_{35,000}$) and outer aqueous phase (MSgg, 5 % $PEG_{35,000}$, 100 mM NaCl, 0.1 % Pluronic F-68) (Figure S19a-c) were chosen. No distinct peaks can be seen in the signal obtained from the unwashed GUVs (Figure S19d), no distinct peaks can be seen and signal processing (Gaussian smoothing and centroiding) possibly removed potential peaks due to a high signal/noise ratio. After washing the GUVs with Milli-Q H_2O twice, a signal increase in the region of interest (blue shaded region) was observed and the peak corresponding to melittin is clearly evident after two washing steps (Figure S19f). In this way, the signal from compounds encapsulated in GUVs was successfully recovered, demonstrating that MALDI-ToF can be used to identify GUV cargoes after repeated washing.

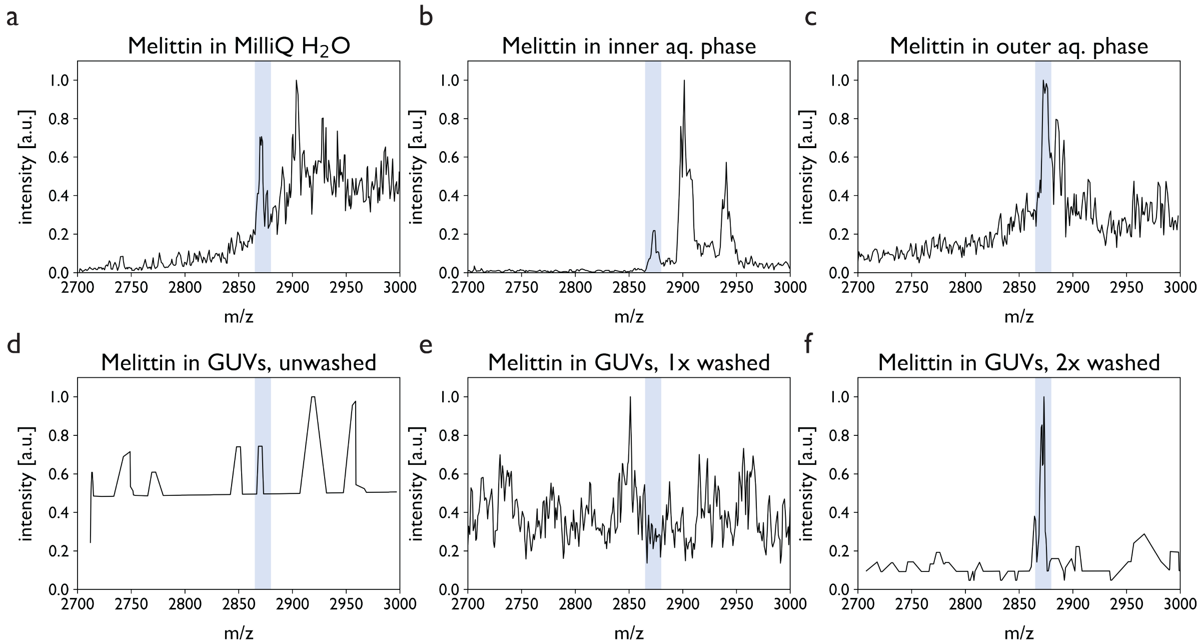

Figure S19: MALDI-ToF MS spectra of melittin in different aqueous buffers: a) Milli-Q H_2O, b) MSgg inner aqueous phase, c) MSgg outer aqueous phase. MALDI-ToF spectra of melittin encapsulated in MSgg-GUVs d) without washing, e) washed 1x with Milli-Q H_2O, and f) washed 2x with Milli-Q H_2O.

3.7 MALDI-ToF Detection of Biofilms in GUVs

In order to determine presence of *B. subtilis* biofilms in GUVs, MALDI-ToF MS spectra of empty GUVs, GUVs containing biofilms and a reference biofilm that was grown on an MSgg agar plate were recorded (Figure S20). Spectra were compared using the Pearson correlation coefficient (Figure 6f). Relevant peaks are highlighted in Figure S20 in blue. In the polymer containing samples (top and middle), peaks with regular spacing ($\sim$ 85 m/z) were detected, corresponding to PMOXA monomers (85 Da) of the PDMS-*b*-PMOXA block copolymer.

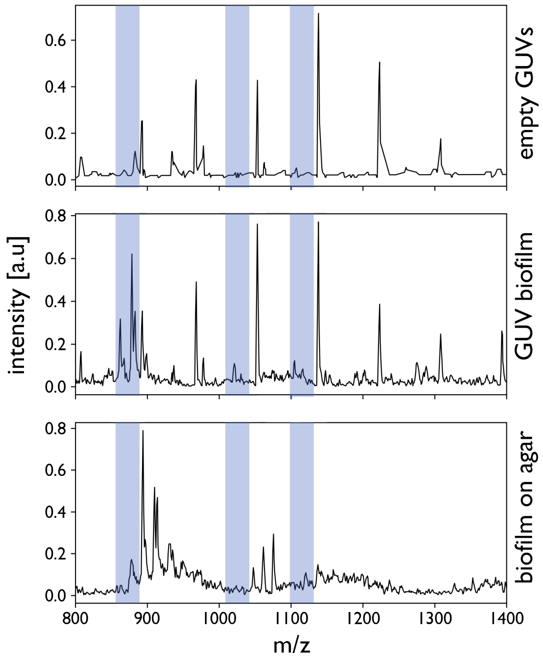

Figure S20: MALDI-ToF MS spectra comparing empty GUVs (top), GUVs containing a *B. subtilis* biofilm (middle) and biofilm samples taken from an MSgg agar plate (bottom). Similar regions highlighted in blue.

3.8 Influence of Long-Term Incubation and Bacterial Presence on Melittin Pores

It is important that GUVs maintain their permeability over time and that it is not compromised by the presence of bacteria. To determine pore localization over time, PDMS-*b*-PMOXA-GUVs permeabilized with 10 μM Cy5-labeled melittin were incubated at 30 °C for 3 days.

Figure S21a shows representative GUVs at day 0 (2 hours after production), day 1, and day 3 of culture. The fluorescence intensity of the Cy5-melittin membrane was measured, and Cy5 fluorescence was found to increase with time (Figure S21a). Fluorescence spectroscopy was used to quantify the number of melittin pores per GUV over time. After incubation with Cy5-melittin, GUVs were washed to remove unbound melittin. The washed GUVs were then lysed by addition of TWEEN20 to a final concentration of 5 % followed by sonication in a water bath for 5 min. The resulting suspensions were expected to contain only the GUV-associated melittin. Using a standard curve with known Cy5-melittin concentration, the Cy5-melittin concentration was determined for the GUV samples. Based on the number concentration of GUVs per sample determined by counting and the concentration of melittin molecules, the number of melittin monomers per GUV was estimated (Figure S21b. The number of melittin pores per GUV was calculated based on the minimum (3) and average (12) number of melittin monomers constituting a pore (Figure S21c).[11–13] This results in 1.5 - 8 x 10^7 pores GUV^{-1} (0.5 - 2 x 10^{-3} pores nm^{-2}) which is in agreement with the numbers of melittin pores found in polymersomes of similar chemical composition (0.6 - 6 x 10^{-3} pores nm^{-2}), accounting for the larger GUV surface area.[11,12] To verify that neither incubation nor the presence of bacteria or biofilms have a negative effect on the affinity of melittin for the membrane, a corresponding experiment in the presence of encapsulated *B. subtilis* was carried out and no major changes in fluorescence intensities measured at the GUV membrane were observed (Figure S21c and d). These results indicate that the membranes of GUV remain permeabilized for the duration of our experiments.

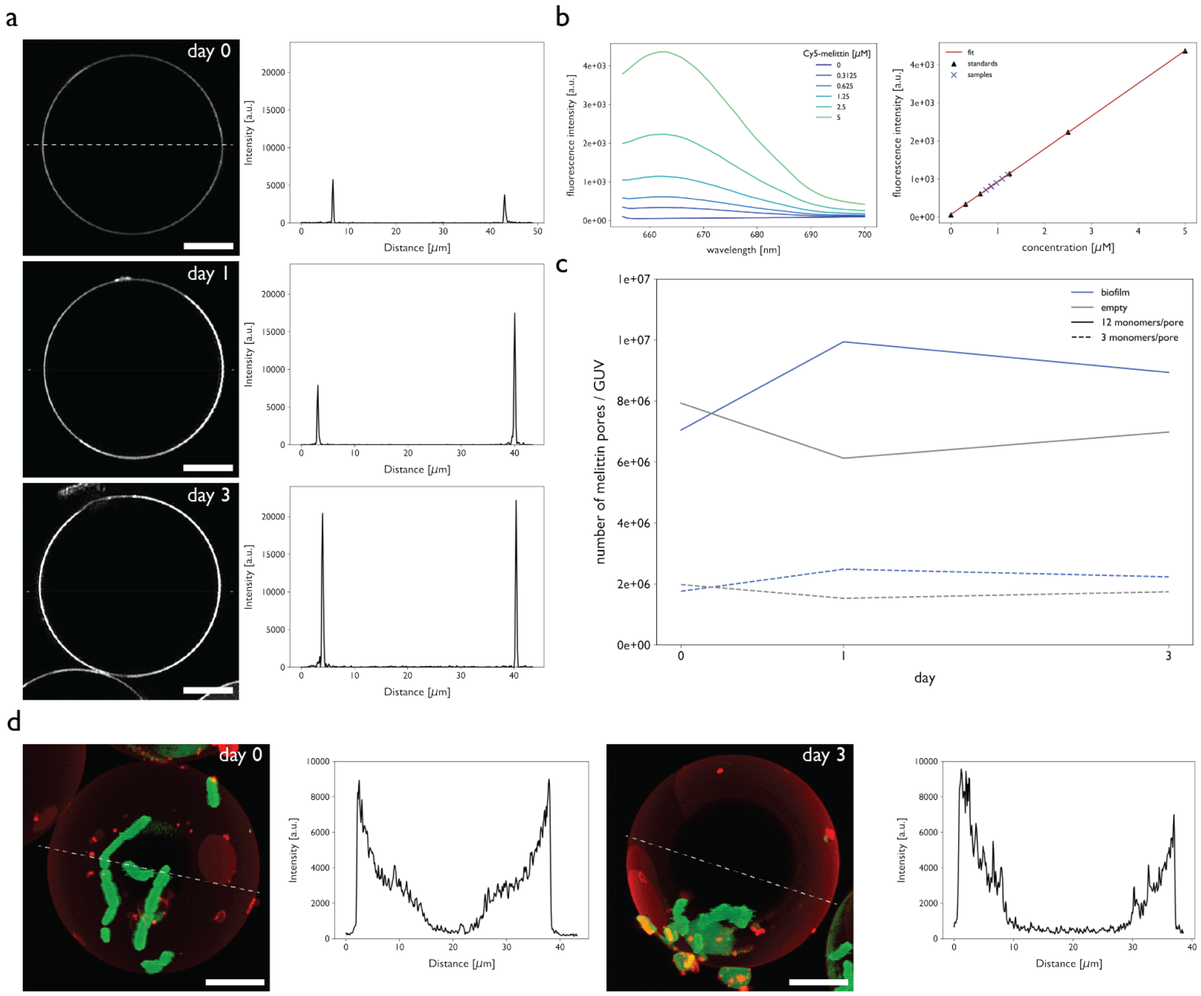

Figure S21: a) Fluorescence micrographs of GUVs permeabilized with Cy5-labeled melittin (white) over time. Profiles show fluorescence signal intensity along the centerline of the depicted GUV sections (dotted line). Scale bars, 10 μm. b) Fluorescence spectra of Cy5-labeled melittin at concentrations of 0-5 μM (left) and standard curve based on the measured fluorescence intensity at the emission maximum (663 nm, black triangles). Linear regression was fitted to the emission data ($R^2 = 0.999$) and used to calculate the Cy5-melittin concentration of the GUV samples (blue crosses). c) Calculated number of pores per GUV based on Cy5-melittin fluorescence intensity, assuming a pore constitution of 3 melittin monomers (dashed lines) or 12 melittin monomers (solid lines) and tested for empty GUVs (gray) and GUVs containing *B. subtilis* (blue) over an incubation time of 3 days at 30 °C. d) Fluorescence micrographs depicting maximal z-projections of *B. subtilis*-encapsulating GUVs permeabilized with Cy5-labeled melittin (red) over the course of 3 days at 30 °C. Profiles were measured along the center line of the depicted GUV z-projections (dashed lines). Scale bars, 10 μm.

3.9 Kanamycin-dependent Killing of Encapsulated *B. subtilis*

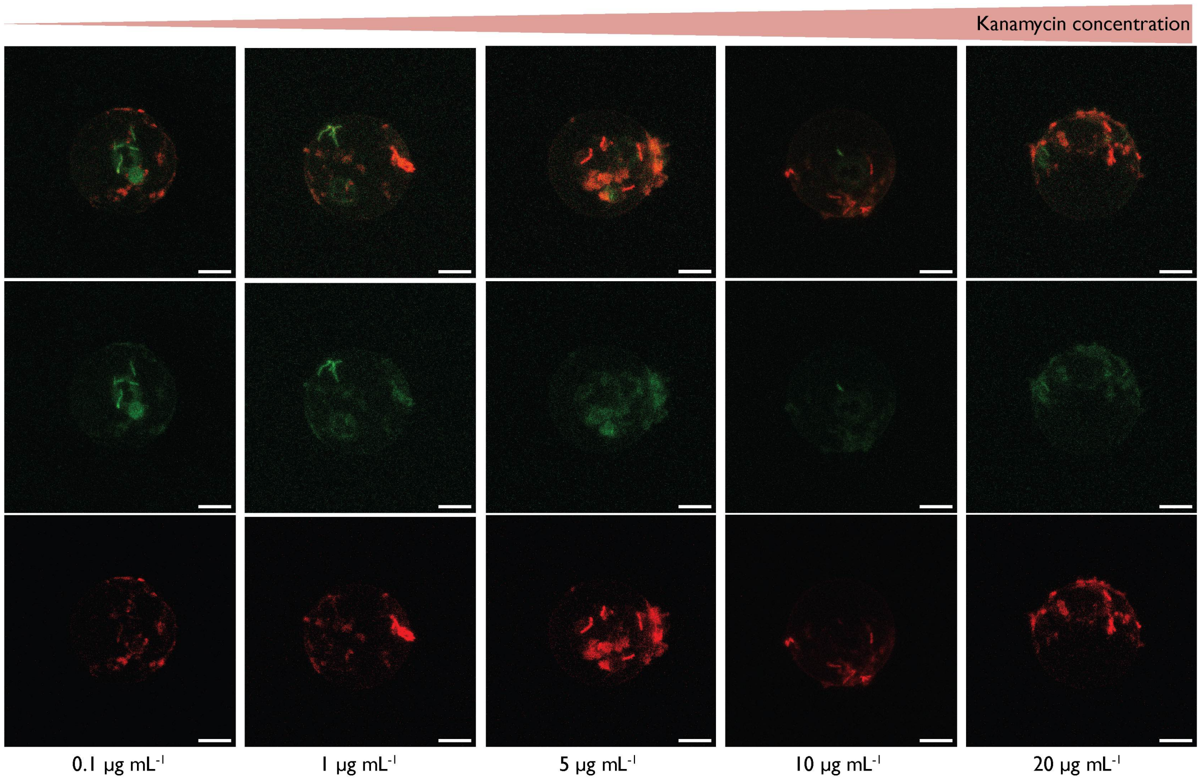

Figure S22: Fluorescent micrographs of polymeric GUVs encapsulating *B. subtilis* (green) stained with propidium iodide (PI, red) in polymeric GUVs with increasing kanamycin concentration. Top row: composite micrographs. Middle row: SYTO 9 bacterial stain single channel micrographs. Bottom row: PI dead stain single channel micrographs. Scale bar, 10 μm.

4 Statistical Analysis

The statistical results from analyses done in this manuscript are presented below. The mean and 95 % confidence interval is plotted and the Tukey HSD post hoc analysis given for each statistical analysis.

4.1 3c) bacteria/GUV

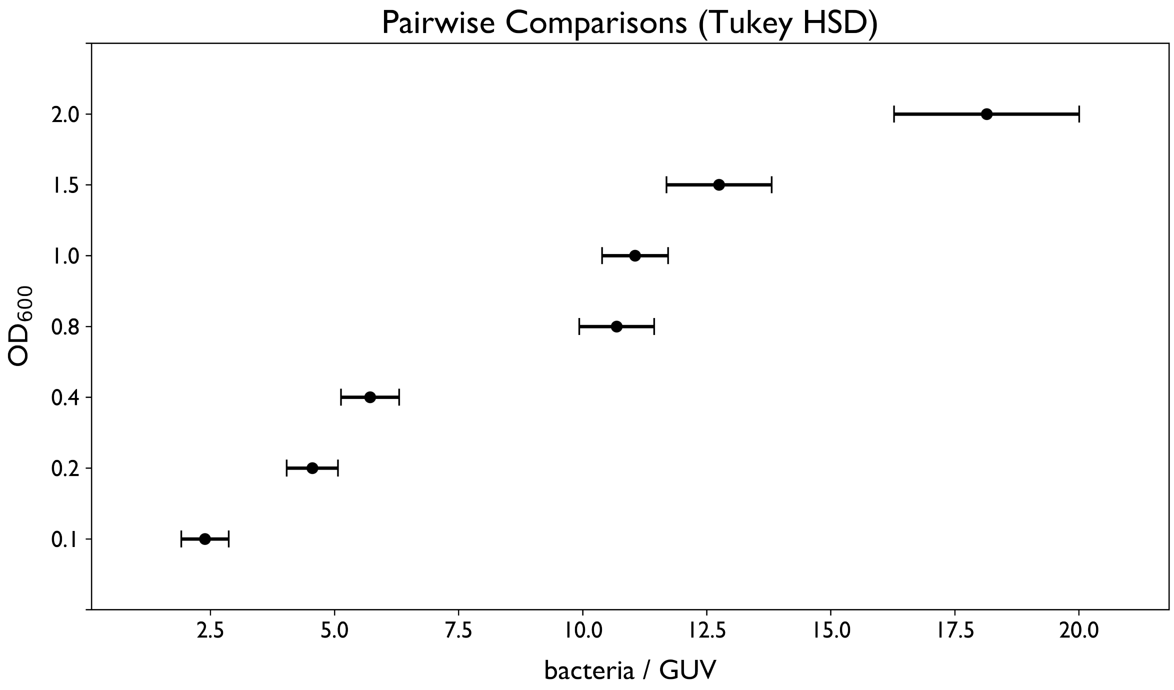

Figure S23: Mean and 95 % confidence interval of number of encapsulated bacteria in GUVs (Figure 3c).

group1	group2	meandiff	p-adj	lower	upper	reject
0.1	0.2	2.1634	0	1.24	3.0867	TRUE
0.1	0.4	3.3261	0	2.3068	4.3454	TRUE
0.1	0.8	8.2971	0	7.0615	9.5328	TRUE
0.1	1	8.668	0	7.5449	9.7912	TRUE
0.1	1.5	10.3606	0	8.7727	11.9484	TRUE
0.1	2	15.7534	0	13.3247	18.1822	TRUE
0.2	0.4	1.1627	0.0234	0.0914	2.234	TRUE
0.2	0.8	6.1338	0	4.8549	7.4126	TRUE
0.2	1	6.5047	0	5.3342	7.6751	TRUE
0.2	1.5	8.1972	0	6.5755	9.8189	TRUE
0.2	2	13.5901	0	11.1391	16.0411	TRUE
0.4	0.8	4.971	0	3.6213	6.3208	TRUE
0.4	1	5.342	0	4.0944	6.5895	TRUE
0.4	1.5	7.0345	0	5.3563	8.7127	TRUE
0.4	2	12.4273	0	9.9386	14.9161	TRUE
0.8	1	0.3709	0.9879	-1.0589	1.8007	FALSE
0.8	1.5	2.0634	0.0145	0.2457	3.8812	TRUE
0.8	2	7.4563	0	4.8714	10.0412	TRUE
1	1.5	1.6925	0.0636	-0.0507	3.4357	FALSE
1	2	7.0854	0	4.5524	9.6184	TRUE
1.5	2	5.3929	0	2.6223	8.1634	TRUE

Table S8: Tukey HSD results of the comparison of the number of encapsulated bacteria in GUVs (Figure 3c).

4.2 5a) melittin influence on growth

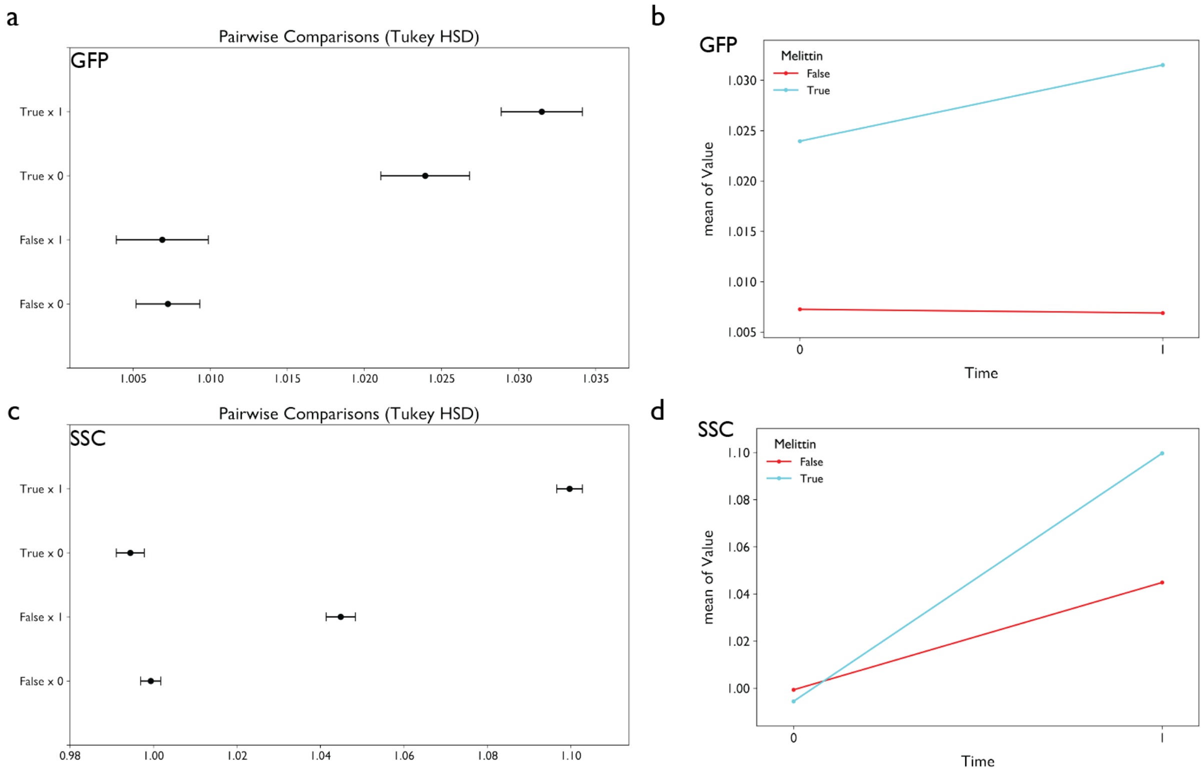

Figure S24: a+c) Mean and 95 % confidence interval of the (a) GFP fluorescence and (c) side scattering (SSC) of GUVs without (False) and with (True) melittin before (0) and after (1) incubation at 37 °C. b+d) Interaction plots of the influence of melittin on the growth of bacteria before (0) and after (1) incubation (Figure 5a)

group1	group2	meandiff	p-adj	lower	upper	reject
False x 0	False x 1	-0.0004	0.9977	-0.0054	0.0047	FALSE
False x 0	True x 0	0.0167	0	0.0117	0.0216	TRUE
False x 0	True x 1	0.0243	0	0.0196	0.0289	TRUE
False x 1	True x 0	0.0171	0	0.0112	0.0229	TRUE
False x 1	True x 1	0.0246	0	0.019	0.0302	TRUE
True x 0	True x 1	0.0076	0.0025	0.002	0.0131	TRUE

Table S9: Tukey HSD results of the comparison of the GFP fluorescence intensities of GUVs with (True) and without (False) melittin before (0) and after (1) incubation at 37 °C (Figure 5a).

group1	group2	meandiff	p-adj	lower	upper	reject
False x 0	False x 1	0.0455	0	0.0396	0.0515	TRUE
False x 0	True x 0	-0.0049	0.1312	-0.0107	0.0009	FALSE
False x 0	True x 1	0.1004	0	0.0949	0.1058	TRUE
False x 1	True x 0	-0.0504	0	-0.0573	-0.0436	TRUE
False x 1	True x 1	0.0548	0	0.0483	0.0614	TRUE
True x 0	True x 1	0.1053	0	0.0988	0.1117	TRUE

Table S10: Tukey HSD results of the comparison of the side scattering intensities of GUVs with (True) and without (False) melittin before (0) and after (1) incubation at 37 °C (Figure 5a).

4.3 6d) biofilm growth in GUVs

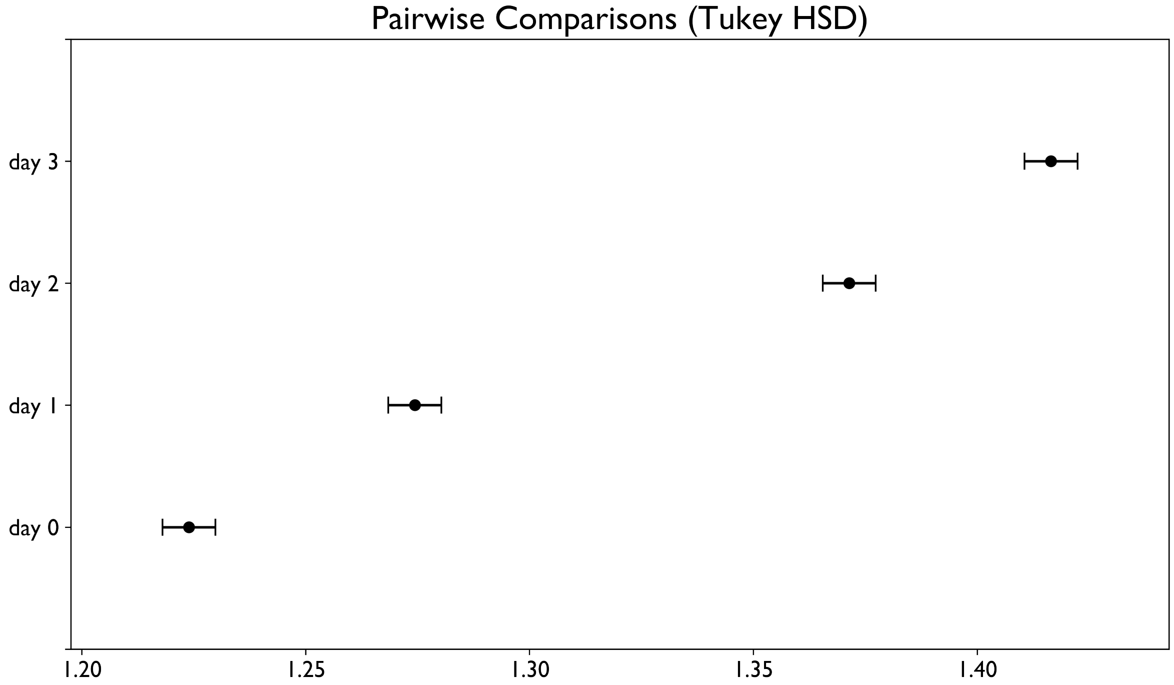

Figure S25: Mean and 95 % confidence interval of the fluorescence intensity (SYPRO Ruby / SYTO 9) of GUVs over time (0-3 days) (Figure 6d).

group1	group2	meandiff	p-adj	lower	upper	reject
day 0	day 1	0.0504	0	0.0386	0.0623	TRUE
day 0	day 2	0.1475	0	0.1356	0.1593	TRUE
day 0	day 3	0.1925	0	0.1807	0.2044	TRUE
day 1	day 2	0.097	0	0.0852	0.1089	TRUE
day 1	day 3	0.1421	0	0.1302	0.1539	TRUE
day 2	day 3	0.0451	0	0.0332	0.0569	TRUE

Table S11: Tukey HSD results of the comparison of the fluorescence intensity (SYPRO Ruby / SYTO 9) of GUVs over time (0-3 days) (Figure 6d).

4.4 6g) antibiotic resistance of biofilm GUVs

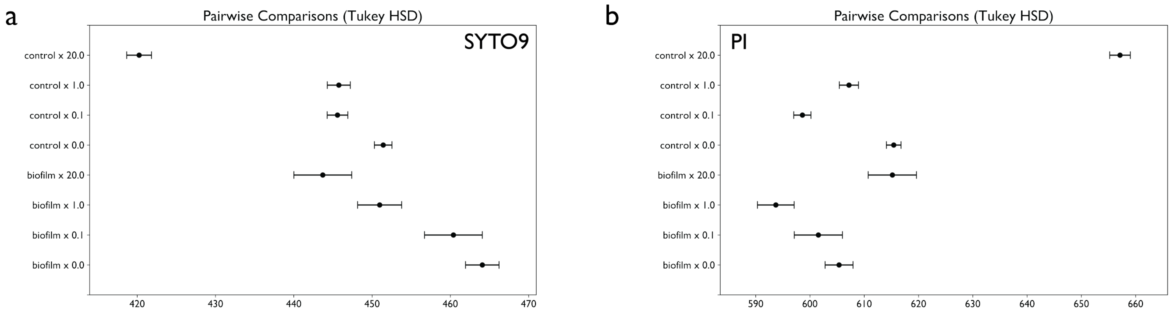

Figure S26: Mean and 95 % confidence interval of a) SYTO 9 and b) PI fluorescence intensity of fresh GUVs (control) or GUVs with bacteria grown inside for 48 h (biofilm) exposed to different antibiotics concentrations (0-20 μg mL^{-1} kanamycin) (Figure 6g).

group1	group2	meandiff	p-adj	lower	upper	reject
biofilm x 0.0	biofilm x 0.1	-3.6941	0.5236	-9.468	2.0797	FALSE
biofilm x 0.0	biofilm x 1.0	-13.1122	0	-18.0522	-8.1723	TRUE
biofilm x 0.0	biofilm x 20.0	-20.3755	0	-26.152	-14.5991	TRUE
biofilm x 0.0	control x 0.0	-12.6611	0	-15.9432	-9.379	TRUE
biofilm x 0.0	control x 0.1	-18.4958	0	-21.9846	-15.0071	TRUE
biofilm x 0.0	control x 1.0	-18.3255	0	-21.9655	-14.6854	TRUE
biofilm x 0.0	control x 20.0	-43.8371	0	-47.5849	-40.0893	TRUE
biofilm x 0.1	biofilm x 1.0	-9.4181	0.0001	-15.6886	-3.1476	TRUE
biofilm x 0.1	biofilm x 20.0	-16.6814	0	-23.63	-9.7328	TRUE
biofilm x 0.1	control x 0.0	-8.967	0	-14.0353	-3.8986	TRUE
biofilm x 0.1	control x 0.1	-14.8017	0	-20.0063	-9.5971	TRUE
biofilm x 0.1	control x 1.0	-14.6313	0	-19.9385	-9.3241	TRUE
biofilm x 0.1	control x 20.0	-40.143	0	-45.5246	-34.7613	TRUE
biofilm x 1.0	biofilm x 20.0	-7.2633	0.0106	-13.5362	-0.9904	TRUE
biofilm x 1.0	control x 0.0	0.4511	1	-3.6421	4.5443	FALSE
biofilm x 1.0	control x 0.1	-5.3836	0.0032	-9.6443	-1.1229	TRUE
biofilm x 1.0	control x 1.0	-5.2132	0.0076	-9.5987	-0.8278	TRUE
biofilm x 1.0	control x 20.0	-30.7249	0	-35.2002	-26.2496	TRUE
biofilm x 20.0	control x 0.0	7.7144	0.0001	2.6431	12.7857	TRUE
biofilm x 20.0	control x 0.1	1.8797	0.9583	-3.3278	7.0871	FALSE
biofilm x 20.0	control x 1.0	2.0501	0.9404	-3.2599	7.3601	FALSE
biofilm x 20.0	control x 20.0	-23.4616	0	-28.846	-18.0771	TRUE
control x 0.0	control x 0.1	-5.8348	0	-7.9613	-3.7082	TRUE
control x 0.0	control x 1.0	-5.6644	0	-8.031	-3.2978	TRUE
control x 0.0	control x 20.0	-31.176	0	-33.7052	-28.6468	TRUE
control x 0.1	control x 1.0	0.1704	1	-2.4754	2.8162	FALSE
control x 0.1	control x 20.0	-25.3413	0	-28.1335	-22.549	TRUE
control x 1.0	control x 20.0	-25.5116	0	-28.4907	-22.5326	TRUE

Table S12: Tukey HSD results of the comparison of the SYTO 9 fluorescence intensity of fresh GUVs (control) or GUVs with bacteria grown inside for 48 h (biofilm) exposed to different antibiotics concentrations (0-20 μg mL^{-1} kanamycin) (Figure 6g).

**group1**	**group2**	**meandiff**	**p-adj**	**lower**	**upper**	**reject**
biofilm x 0.0	biofilm x 0.1	-3.8135	0.7104	-10.7577	3.1307	FALSE
biofilm x 0.0	biofilm x 1.0	-11.6569	0	-17.5982	-5.7156	TRUE
biofilm x 0.0	biofilm x 20.0	9.8111	0.0005	2.8638	16.7584	TRUE
biofilm x 0.0	control x 0.0	10.064	0	6.1166	14.0114	TRUE
biofilm x 0.0	control x 0.1	-6.7612	0	-10.9572	-2.5653	TRUE
biofilm x 0.0	control x 1.0	1.8137	0.9149	-2.5642	6.1916	FALSE
biofilm x 0.0	control x 20.0	51.7563	0	47.2488	56.2638	TRUE
biofilm x 0.1	biofilm x 1.0	-7.8434	0.0347	-15.3849	-0.3018	TRUE
biofilm x 0.1	biofilm x 20.0	13.6246	0	5.2675	21.9817	TRUE
biofilm x 0.1	control x 0.0	13.8775	0	7.7818	19.9732	TRUE
biofilm x 0.1	control x 0.1	-2.9477	0.8448	-9.2073	3.3118	FALSE
biofilm x 0.1	control x 1.0	5.6273	0.1311	-0.7557	12.0102	FALSE
biofilm x 0.1	control x 20.0	55.5698	0	49.0973	62.0424	TRUE
biofilm x 1.0	biofilm x 20.0	21.468	0	13.9236	29.0124	TRUE
biofilm x 1.0	control x 0.0	21.7209	0	16.798	26.6438	TRUE
biofilm x 1.0	control x 0.1	4.8957	0.0734	-0.2287	10.02	FALSE
biofilm x 1.0	control x 1.0	13.4706	0	8.1963	18.745	TRUE
biofilm x 1.0	control x 20.0	63.4132	0	58.0308	68.7956	TRUE
biofilm x 20.0	control x 0.0	0.2529	1	-5.8463	6.3522	FALSE
biofilm x 20.0	control x 0.1	-16.5723	0	-22.8353	-10.3093	TRUE
biofilm x 20.0	control x 1.0	-7.9974	0.0037	-14.3837	-1.611	TRUE
biofilm x 20.0	control x 20.0	41.9452	0	35.4694	48.4211	TRUE
control x 0.0	control x 0.1	-16.8252	0	-19.3829	-14.2676	TRUE
control x 0.0	control x 1.0	-8.2503	0	-11.0966	-5.4039	TRUE
control x 0.0	control x 20.0	41.6923	0	38.6504	44.7342	TRUE
control x 0.1	control x 1.0	8.575	0	5.3929	11.7571	TRUE
control x 0.1	control x 20.0	58.5176	0	55.1594	61.8758	TRUE
control x 1.0	control x 20.0	49.9426	0	46.3597	53.5255	TRUE

Table S13: Tukey HSD results of the comparison of the PI fluorescence intensity of fresh GUVs (control) or GUVs with bacteria grown inside for 48 h (biofilm) exposed to different antibiotics concentrations (0-20 μg mL^{-1} kanamycin) (Figure 6g).

4.5 7) antibiotic screening of bacteria-GUVs

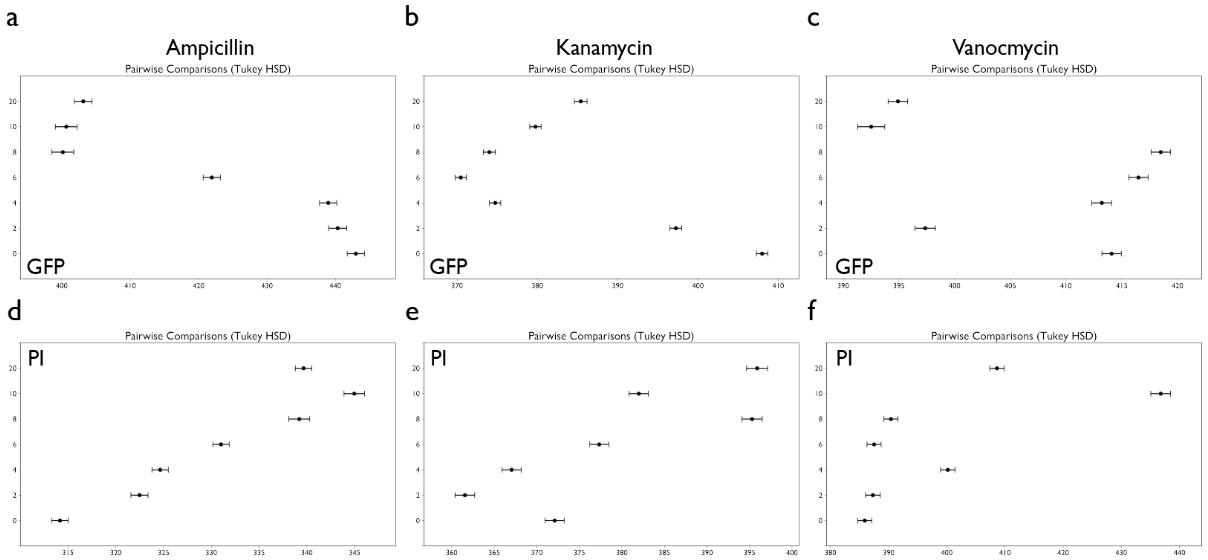

Figure S27: Mean and 95 % confidence interval of a-c) GFP and d-f) PI fluorescence intensity of *E. coli*-encapsulating GUVs exposed to different concentrations (0-20 μg mL^{-1}) of the antibiotics a+d) ampicillin, b+e) kanamycin, and c+f) vancomycin (Figure 7).

4.6 7b) Ampicillin

group1	group2	meandiff	p-adj	lower	upper	reject
0	2	-2.6489	0.0003	-5.242	-0.0558	TRUE
0	4	-4.0395	0	-6.5658	-1.5131	TRUE
0	6	-21.0593	0	-23.5856	-18.5329	TRUE
0	8	-42.8388	0	-45.729	-39.9486	TRUE
0	10	-42.3415	0	-45.1932	-39.4898	TRUE
0	20	-39.8565	0	-42.3922	-37.3208	TRUE
2	4	-1.3906	0.2637	-3.9837	1.2025	FALSE
2	6	-18.4104	0	-21.0035	-15.8173	TRUE
2	8	-40.1899	0	-43.1387	-37.2411	TRUE
2	10	-39.6926	0	-42.6036	-36.7816	TRUE
2	20	-37.2076	0	-39.8098	-34.6054	TRUE
4	6	-17.0198	0	-19.5461	-14.4934	TRUE
4	8	-38.7993	0	-41.6895	-35.9091	TRUE
4	10	-38.302	0	-41.1537	-35.4503	TRUE
4	20	-35.817	0	-38.3527	-33.2814	TRUE
6	8	-21.7795	0	-24.6697	-18.8893	TRUE
6	10	-21.2822	0	-24.1339	-18.4306	TRUE
6	20	-18.7972	0	-21.3329	-16.2616	TRUE
8	10	0.4973	0.9947	-2.6812	3.6758	FALSE
8	20	2.9823	0.0003	0.0839	5.8806	TRUE
10	20	2.485	0.0047	-0.3749	5.3449	FALSE

Table S14: Tukey HSD results of the comparison of the GFP fluorescence intensity of *E. coli*-encapsulating GUVs exposed to different concentrations (0-20 μg mL^{-1}) of the antibiotic ampicillin (Figure 7).

group1	group2	meandiff	p-adj	lower	upper	reject
0	2	8.3263	0	6.5722	10.0803	TRUE
0	4	10.4914	0	8.7826	12.2003	TRUE
0	6	16.8584	0	15.1496	18.5673	TRUE
0	8	25.0469	0	23.0919	27.0019	TRUE
0	10	30.7981	0	28.8691	32.727	TRUE
0	20	25.4966	0	23.7814	27.2118	TRUE
2	4	2.1652	0	0.4111	3.9192	TRUE
2	6	8.5322	0	6.7781	10.2862	TRUE
2	8	16.7206	0	14.726	18.7152	TRUE
2	10	22.4718	0	20.5027	24.4409	TRUE
2	20	17.1703	0	15.4102	18.9305	TRUE
4	6	6.367	0	4.6581	8.0759	TRUE
4	8	14.5554	0	12.6004	16.5104	TRUE
4	10	20.3066	0	18.3777	22.2356	TRUE
4	20	15.0052	0	13.29	16.7203	TRUE
6	8	8.1884	0	6.2334	10.1434	TRUE
6	10	13.9396	0	12.0107	15.8686	TRUE
6	20	8.6382	0	6.923	10.3533	TRUE
8	10	5.7512	0	3.6012	7.9012	TRUE
8	20	0.4497	0.9611	-1.5108	2.4102	FALSE
10	20	-5.3015	0	-7.236	-3.3669	TRUE

Table S15: Tukey HSD results of the comparison of the PI fluorescence intensity of *E. coli*-encapsulating GUVs exposed to different concentrations (0-20 μg mL^{-1}) of the antibiotic ampicillin (Figure 7).

4.6.1 7c) Kanamycin

group1	group2	meandiff	p-adj	lower	upper	reject
0	2	-3.3633	0	-5.1415	-1.5851	TRUE
0	4	-8.7799	0	-10.5118	-7.048	TRUE
0	6	-13.8792	0	-15.6388	-12.1195	TRUE
0	8	-18.0672	0	-19.7991	-16.3353	TRUE
0	10	-12.2283	0	-13.9602	-10.4964	TRUE
0	20	7.4096	0	5.6576	9.1616	TRUE
2	4	-5.4166	0	-7.1948	-3.6384	TRUE
2	6	-10.5159	0	-12.3211	-8.7106	TRUE
2	8	-14.7039	0	-16.4821	-12.9257	TRUE
2	10	-8.865	0	-10.6432	-7.0868	TRUE
2	20	10.7729	0	8.9751	12.5707	TRUE
4	6	-5.0993	0	-6.859	-3.3396	TRUE
4	8	-9.2873	0	-11.0192	-7.5554	TRUE
4	10	-3.4484	0	-5.1803	-1.7165	TRUE
4	20	16.1895	0	14.4374	17.9415	TRUE
6	8	-4.188	0	-5.9477	-2.4284	TRUE
6	10	1.6509	0.0015	-0.1088	3.4105	FALSE
6	20	21.2888	0	19.5093	23.0682	TRUE
8	10	5.8389	0	4.107	7.5708	TRUE
8	20	25.4768	0	23.7248	27.2288	TRUE
10	20	19.6379	0	17.8859	21.3899	TRUE

Table S16: Tukey HSD results of the comparison of the GFP fluorescence intensity of *E. coli*-encapsulating GUVs exposed to different concentrations (0-20 μg mL^{-1}) of the antibiotic kanamycin (Figure 7).

group1	group2	meandiff	p-adj	lower	upper	reject
0	2	-1.67	0	-3.1485	-0.1916	TRUE
0	4	-9.4487	0	-10.8887	-8.0088	TRUE
0	6	-0.2128	0.9986	-1.6758	1.2503	FALSE
0	8	-8.394	0	-9.834	-6.9541	TRUE
0	10	-13.673	0	-15.1129	-12.233	TRUE
0	20	-20.3285	0	-21.7852	-18.8718	TRUE
2	4	-7.7787	0	-9.2572	-6.3002	TRUE
2	6	1.4573	0.0009	-0.0437	2.9582	FALSE
2	8	-6.724	0	-8.2025	-5.2455	TRUE
2	10	-12.0029	0	-13.4814	-10.5245	TRUE
2	20	-18.6584	0	-20.1532	-17.1637	TRUE
4	6	9.236	0	7.7729	10.699	TRUE
4	8	1.0547	0.0368	-0.3853	2.4947	FALSE
4	10	-4.2242	0	-5.6642	-2.7842	TRUE
4	20	-10.8797	0	-12.3364	-9.423	TRUE
6	8	-8.1813	0	-9.6443	-6.7182	TRUE
6	10	-13.4602	0	-14.9233	-11.9971	TRUE
6	20	-20.1157	0	-21.5952	-18.6362	TRUE
8	10	-5.2789	0	-6.7189	-3.8389	TRUE
8	20	-11.9344	0	-13.3911	-10.4777	TRUE
10	20	-6.6555	0	-8.1122	-5.1988	TRUE

Table S17: Tukey HSD results of the comparison of the PI fluorescence intensity of *E. coli*-encapsulating GUVs exposed to different concentrations (0-20 μg mL^{-1}) of the antibiotic kanamycin (Figure 7).

4.6.2 7d) Vancomycin

group1	group2	meandiff	p-adj	lower	upper	reject
0	2	-16.7204	0	-18.5019	-14.9388	TRUE
0	4	-0.8788	0.3484	-2.6365	0.8789	FALSE
0	6	2.4132	0	0.6766	4.1498	TRUE
0	8	4.4152	0	2.6743	6.1561	TRUE
0	10	-21.5641	0	-23.6503	-19.478	TRUE
0	20	-19.1704	0	-20.9195	-17.4214	TRUE
2	4	15.8416	0	14.0408	17.6424	TRUE
2	6	19.1336	0	17.3534	20.9139	TRUE
2	8	21.1356	0	19.3511	22.92	TRUE
2	10	-4.8438	0	-6.9664	-2.7211	TRUE
2	20	-2.4501	0	-4.2425	-0.6577	TRUE
4	6	3.292	0	1.5356	5.0484	TRUE
4	8	5.294	0	3.5333	7.0546	TRUE
4	10	-20.6854	0	-22.788	-18.5827	TRUE
4	20	-18.2917	0	-20.0604	-16.523	TRUE
6	8	2.002	0	0.2624	3.7416	TRUE
6	10	-23.9774	0	-26.0624	-21.8923	TRUE
6	20	-21.5837	0	-23.3314	-19.836	TRUE
8	10	-25.9794	0	-28.068	-23.8907	TRUE
8	20	-23.5857	0	-25.3377	-21.8336	TRUE
10	20	2.3937	0	0.2983	4.4891	TRUE

Table S18: Tukey HSD results of the comparison of the GFP fluorescence intensity of *E. coli*-encapsulating GUVs exposed to different concentrations (0-20 μg mL^{-1}) of the antibiotic vancomycin (Figure 7).

group1	group2	meandiff	p-adj	lower	upper	reject
0	2	1.4053	0.2033	-1.0741	3.8847	FALSE
0	4	14.2757	0	11.8295	16.7219	TRUE
0	6	1.6071	0.0751	-0.8098	4.0239	FALSE
0	8	4.5003	0	2.0775	6.9232	TRUE
0	10	50.8663	0	47.963	53.7696	TRUE
0	20	22.729	0	20.2949	25.1632	TRUE
2	4	12.8704	0	10.3642	15.3766	TRUE
2	6	0.2018	0.9999	-2.2758	2.6793	FALSE
2	8	3.095	0	0.6116	5.5785	TRUE
2	10	49.461	0	46.5069	52.4151	TRUE
2	20	21.3237	0	18.8292	23.8182	TRUE
4	6	-12.6686	0	-15.113	-10.2243	TRUE
4	8	-9.7754	0	-12.2257	-7.3251	TRUE
4	10	36.5906	0	33.6643	39.5169	TRUE
4	20	8.4533	0	5.9919	10.9148	TRUE
6	8	2.8933	0	0.4723	5.3142	TRUE
6	10	49.2592	0	46.3575	52.161	TRUE
6	20	21.122	0	18.6896	23.5543	TRUE
8	10	46.366	0	43.4592	49.2728	TRUE
8	20	18.2287	0	15.7904	20.667	TRUE
10	20	-28.1373	0	-31.0535	-25.221	TRUE

Table S19: Tukey HSD results of the comparison of the PI fluorescence intensity of *E. coli*-encapsulating GUVs exposed to different concentrations (0-20 μg mL^{-1}) of the antibiotic vancomycin (Figure 7).

References

[1] E. C. dos Santos, A. Angelini, D. Hürlimann, W. Meier, C. G. Palivan, *Chemistry* **2020**, *2*, 2 470.

[2] R. Lambert, J. Pearson, *Journal of Applied Microbiology* **2000**, *88*, 5 784.

[3] R. Weishaupt, L. Heuberger, G. Siqueira, B. Gutt, T. Zimmermann, K. Maniura-Weber, S. Salentinig, G. Faccio, *ACS Applied Materials & Interfaces* **2018**, *10*, 23 20170.

[4] A. DeLean, P. J. Munson, D. Rodbard, *American Journal of Physiology-Endocrinology and Metabolism* **1978**, *235*, 2 E97.

[5] C. E. Meyer, I. Craciun, C.-A. Schoenenberger, R. Wehr, C. G. Palivan, *Nanoscale* **2021**, *13*, 1 66.

[6] A. Peyret, E. Ibarboure, J.-F. Le Meins, S. Lecommandoux, *Advanced Science* **2018**, *5*, 1 1700453.

[7] P. Dwivedi, G. Puzon, M. Tam, D. Langlais, S. Jackson, K. Kaplan, W. F. Siems, A. J. Schultz, L. Xun, A. Woods, H. H. Hill, *Journal of Mass Spectrometry* **2010**, *45*, 12 1383.

[8] C. G. Clark, P. Kruczkiewicz, C. Guan, S. J. McCorrister, P. Chong, J. Wylie, P. van Caeseele, H. A. Tabor, P. Snarr, M. W. Gilmour, E. N. Taboada, G. R. Westmacott, *Journal of Microbiological Methods* **2013**, *94*, 3 180.

[9] A. Croxatto, G. Prod'hom, G. Greub, *FEMS Microbiology Reviews* **2012**, *36*, 2 380.

[10] J. Chandler, C. Haslam, N. Hardy, M. Leveridge, P. Marshall, *SLAS DISCOVERY: Advancing the Science of Drug Discovery* **2017**, *22*, 10 1262.

[11] M. Korpidou, V. Maffeis, I. A. Dinu, C.-A. Schoenenberger, W. P. Meier, C. G. Palivan, *Journal of Materials Chemistry B* **2022**, *10*, 20 3916.

[12] A. Belluati, V. Mikhalevich, S. Yorulmaz Avsar, D. Daubian, I. Craciun, M. Chami, W. P. Meier, C. G. Palivan, *Biomacromolecules* **2020**, *21*, 2 701.

[13] J. H. Lin, A. Baumgaertner, *Biophysical Journal* **2000**, *78*, 4 1714.
